# Predictomes, a classifier-curated database of AlphaFold-modeled protein-protein interactions

**DOI:** 10.1016/j.molcel.2025.01.034

**Published:** 2025-02-26

**Authors:** Ernst W. Schmid, Johannes C. Walter

**Affiliations:** 1 Department of Biological Chemistry & Molecular Pharmacology, Blavatnik Institute, Harvard Medical School, Boston, MA 02115, USA; 2 Howard Hughes Medical Institute, Boston, MA 02115, USA

## Abstract

Protein-protein interactions (PPIs) are ubiquitous in biology, yet a comprehensive structural characterization of the PPIs underlying cellular processes is lacking. AlphaFold-Multimer (AF-M) has the potential to fill this knowledge gap, but standard AF-M confidence metrics do not reliably separate relevant PPIs from an abundance of false positive predictions. To address this limitation, we used machine learning on curated datasets to train a Structure Prediction and Omics informed Classifier (SPOC) that effectively separates true and false AF-M predictions of PPIs, including in proteome-wide screens. We applied SPOC to an all-by-all matrix of nearly 300 human genome maintenance proteins, generating ~40,000 predictions that can be viewed at predictomes.org, where users can also score their own predictions with SPOC. High confidence PPIs discovered using our approach power hypothesis generation in genome maintenance. Our results provide a framework for interpreting large scale AF-M screens and help lay the foundation for a proteome-wide structural interactome.

## eTOC

Schmid and Walter train a classifier that discerns functionally relevant structure predictions in proteome-wide protein-protein interaction (PPI) screens using AlphaFold-Multimer, and they use this confidence metric to curate a database of 40,000 predicted interactions among ~300 genome maintenance proteins.

## Graphical Abstract

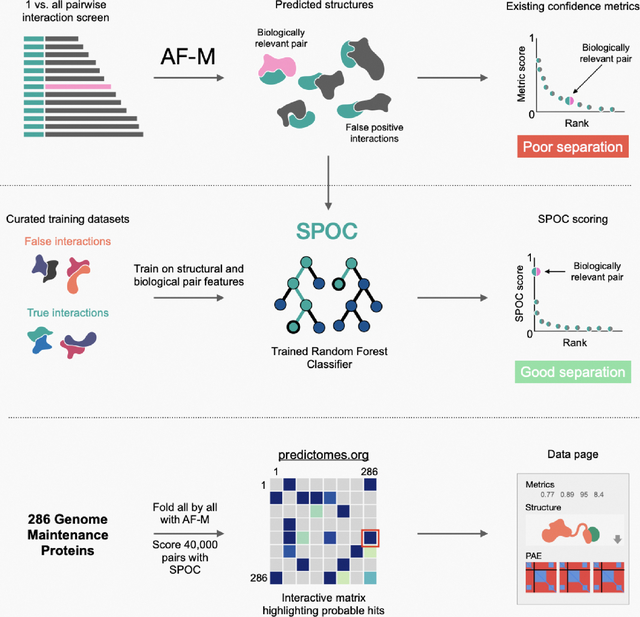

## Introduction

Most biological processes depend on the interaction of multiple proteins^1^. Stable protein-protein interactions (PPIs) form large cellular structures (e.g. the nuclear pore) and stable molecular machines (e.g. RNA polymerase), whereas transient interactions underlie dynamic processes ranging from signaling to DNA replication. The ~20,000 proteins encoded by the human genome can theoretically combine in ~200 million binary combinations, but current estimates suggest that only ~1.5 million pairings represent functional PPIs^2^. Of these, only 50,000 (3%) have been identified^2^, and ~9,000 (0.5%) are structurally resolved. These estimates, though necessarily imprecise, indicate that most PPIs are both unknown and structurally inaccessible. Recognizing this knowledge gap, investigators have long sought to discover PPIs at scale using experimental and computational approaches^3^. Experimental approaches toward this goal include yeast two hybrid assays^2,4^, co-immunoprecipitation^5^, column chromatography based complex fractionation^6^, and cross-linking coupled with mass spectrometry^7^. Computational strategies include homology modeling^8,9^, rigid body docking^10^, and linking genomic data with structural information^11^. While these methods have uncovered many PPIs, they are laborious, yield many false positive and false negative interactions, and thus far have not generated a comprehensive structural interactome.

To address these challenges, researchers are increasingly using deep learning methods to model protein structures^12,13^ and PPIs. The most popular predictive algorithm is AlphaFold-multimer (AF-M)^12^, a deep neural network that uses similar principles as AlphaFold to predict structures of multi-chain complexes. AF-M was trained using five distinct regimens with structures from the Protein Data Bank (PDB^14^), yielding five models, each of which makes a structure prediction. AF-M is being used to uncover PPIs on the scale of pathways and organisms^15–24^. While many studies have examined AF-M’s ability to correctly predict structures in curated PPI databases^25–27^, there has been less focus on separating true from false interactions in large-scale unbiased screens. In addition, while various groups have proposed different metrics for evaluating interface prediction quality and confidence^28^, there has to our knowledge not been a systematic effort to compare these metrics on a single, unbiased dataset or integrate them into a combined and potentially better performing metascore.

We previously used *in silico* screening with AF-M to uncover how the protein DONSON promotes replication initiation^29^. Folding DONSON with 70 core replication proteins and quantifying the agreement among the five AF-M models (a metric we call average models or “avg_models”) identified five functional DONSON-interacting proteins^30–32^. In the first proteome-wide AF-M screen, we also folded DONSON with over 20,000 human proteins and scored the results using avg_models and other AF-M confidence metrics (e.g. ipTM, pDockQ)^33^. However, in this case, DONSON’s functional partners were distributed over the top hundreds or even thousands of hits. Given the poor performance of existing metrics in this proteome-wide screen, we sought to develop a more robust scoring system that successfully identifies true PPIs among interacting pairs that are predicted by AF-M.

In this study, we systematically assessed AF-M’s ability to recover true PPIs embedded in a large set of decoy interactions. This analysis showed that standard metrics indeed perform poorly in identifying true interactions. We then used machine learning to train a classifier on a curated set of positive and negative AF-M predictions of binary complexes. This classifier considers structural and biological features of the pairwise predictions and is called SPOC (Structure Prediction and Omics-based Classifier). SPOC outperforms standard metrics in separating positive and negative predictions, including in several proteome-wide *in silico* screens. We further applied SPOC to an all-by-all interaction matrix of 286 human genome maintenance proteins (~40,000 pairs), leading to the identification of many high confidence predictions. These can be viewed and downloaded at predictomes.org, where users can also score their own predictions with SPOC. In summary, our study introduces SPOC, which guides interpretation of large scale *in silico* screens, and reports a user-friendly web interface with large-scale structure predictions in the genome maintenance field that drive hypothesis generation.

## Results

### Canonical confidence metrics are inadequate to evaluate large-scale AF-M screens

The results of PPI screens using AF-M are typically ranked using metrics such as the interface predicted Template Modeling score (ipTM, 0–1 scale; >0.5 is considered confident), AF-M’s estimate of interface accuracy^12^. Another common metric is pDockQ^34^, which considers the predicted number of interacting residues and their local positioning confidence, pLDDT (predicted Local Distance Difference Test) (0 – 1 scale; >0.23 is confident). Because pDockQ and ipTM scores can be high for structures that contain spurious interfaces^14,31^, we previously developed a metric that also considers another AF-M output, the predicted alignment error (PAE; 0–30 Å scale, lower is better), a measure of AF-M’s confidence in the *global* positioning of residues, including across interfaces. Specifically, we filter AF-M predictions to identify those in which at least five interfacial residue pairs have a PAE value <15 Å. In addition, both residues in each pair must have pLDDT values >50 and reside between 1Å and 5Å of each other (Figure S1A). Only these “contact positive” (“C+”) pairs are subject to downstream analysis. We then quantify how well the independently-trained AF-M models agree on the position of all C+ pairs, generating an “average models” (avg_models) score^33^ (Figure S1B; 0–1 scale, >0.5 is confident and means more than half the models used during inference agree on all C+ contacts). Although this metric was useful in scoring a small-scale *in silico* screen, its performance was poor in a proteome-wide screen^33^.

**Figure 1:**
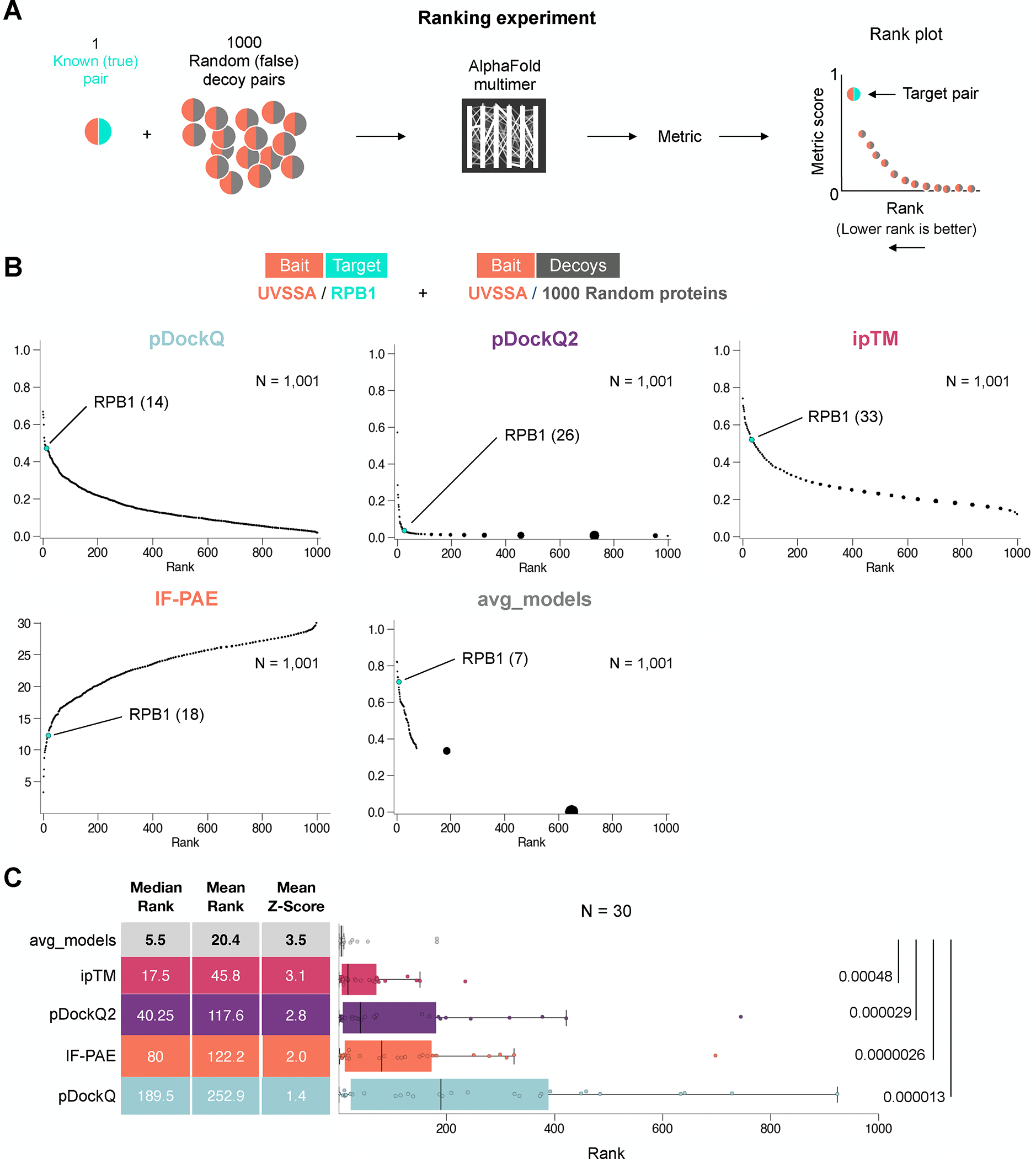
Current metrics are inadequate to evaluate large-scale AF-M screens **(A)** Schematic of a “ranking experiment” to evaluate AF-M prediction quality metrics. **(B)** Example of a ranking experiment where the target pair UVSSA/RPB1 was embedded in a set of 1,000 decoy (UVSSA + random) pairs and evaluated using five different metrics. **(C)** Box plots comparing the performance of five different metrics across 30 different ranking experiments. Lines indicate medians and boxes span the first to third quartile. P-values shown are from Wilcoxon signed-rank tests of all metrics compared to avg_models.

To systematically measure the performance of various confidence metrics in large-scale *in silico* screening, we assessed their ability to rank a single functional interaction ahead of many interactions that are likely false (“Ranking” experiment; Figure 1A). To this end, we identified 30 well-characterized protein complexes that were not in the PDB and therefore could not have informed AF-M (Table S1). The only exception was UVSSA-RPB1, whose structure was published after the training cutoff for AF-M v3, which was used for our experiments^35^. We considered these pairs to be positives (P). For each pair, one protein was selected as “bait” and paired with 1,000 different and randomly selected prey proteins, the vast majority of which should be negative (N). To reduce computation time and maximize throughput, all pairs were folded in three out of five AF-M models (3 recycles; templates enabled). For example, the bait protein UVSSA was folded with its known partner RPB1 and with 1,000 random prey proteins. The resulting structures were ranked by pDockQ, pDockQ2^36^, ipTM and avg_models. We additionally ranked structures by IF-PAE^27^ by identifying interface residue pairs (<5 Å distance) and averaging their PAE values. The results showed that avg_models performed best, giving UVSSA/RPB1 the highest rank, 7^th^ out of 1,001 pairs (Figure 1B). In 30 independent ranking experiments, avg_models consistently outperformed all other metrics (Figure 1C). However, in proteome-wide screens, where the number of negatives should be ~20 times higher, even the avg_models metric would be expected to mix the positive pair with 100–200 random interactions. Indeed, although avg_models performed best in ranking DONSON’s true interactors in a previous proteome-wide screen^23^, they were still mixed in with hundreds of other proteins, the vast majority of which are presumably false positives (Figure S2A–C)^33^. These results show that existing confidence metrics are insufficient to identify true PPIs in large scale *in silico* interaction screens.

### Curated sets of PPIs for classifier training

We sought to use machine learning to train an algorithm or “classifier” that can accurately score and rank AF-M predictions. For training and evaluation, we curated five datasets corresponding to true (biologically meaningful) and negative (likely false or spurious) pairs. To avoid confounding effects ^37^ of training on positive pairs whose interaction is not detected by AF-M, we included only contact positive (C+) pairs in our training sets.

**Figure 2:**
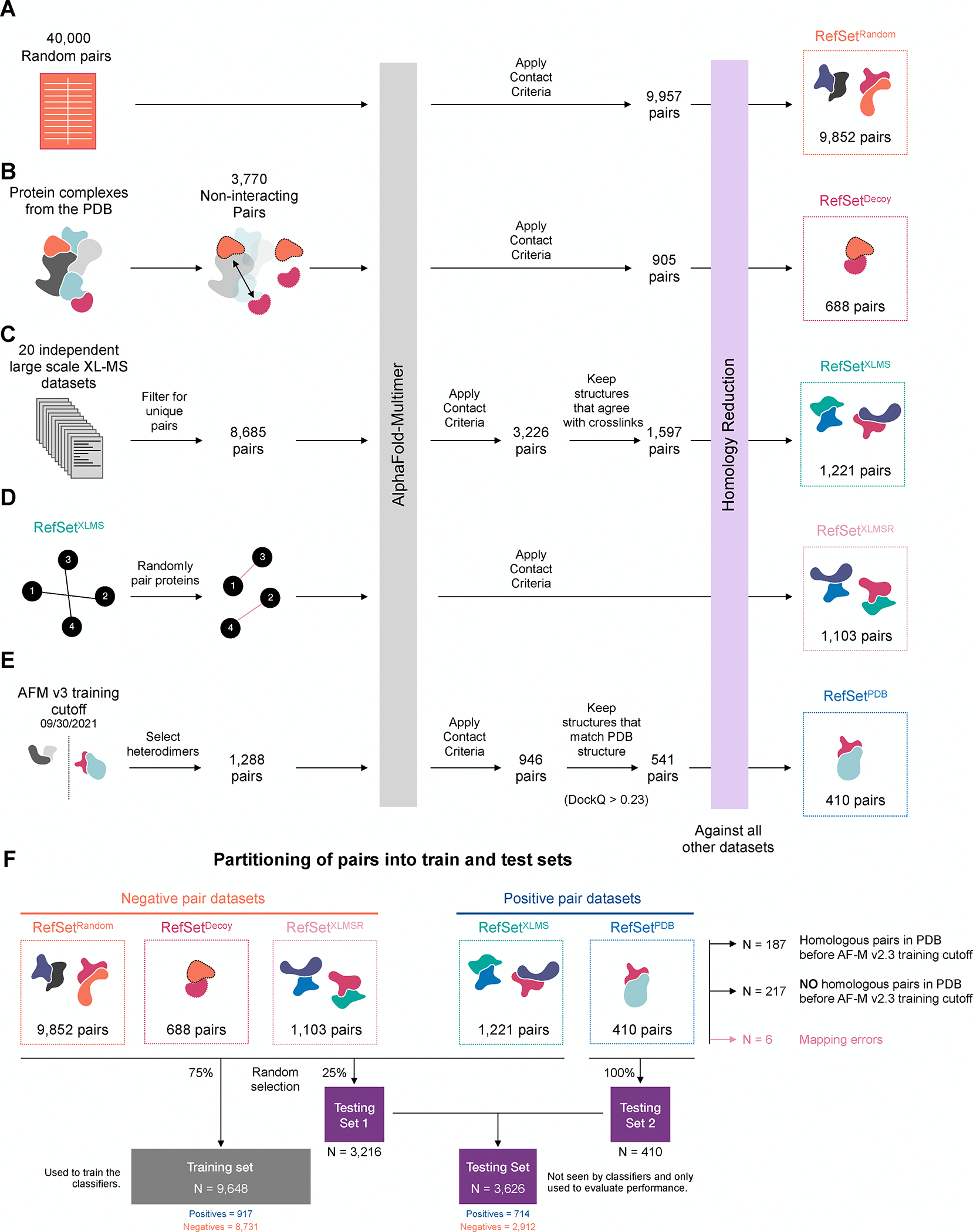
Assembling curated sets for classifier training **(A)** Schematic illustrating the methodology for constructing the random pairing reference set (RefSet^Random^). We generated 40,000 random pairs by repeatedly sampling from the canonical human protein entries in UniProt. Pairs that exceeded 3,600 amino acids (GPU memory limit) or that were present in another dataset were discarded. As a final step, homology reduction was performed across all 5 datasets to ensure no two pairs are homologous**. (B)** Schematic illustrating assembly of the PDB decoy set (RefSet^Decoy^). We selected protein pairs that do not make direct contact (defined as more than 10 residue pairs with heavy(non-hydrogen) atoms closer than 5Å) in large multi-subunit complexes of known structure. **(C)** Schematic illustrating assembly of the (RefSet^XLMS^) set, which was mined from human or mouse cross linking datasets. **(D)** Schematic illustrating assembly of the (RefSet^XLMSR^) set that was constructed by randomly shuffling and pairing proteins from the XLMS set. After repeated sampling and AF-M folding the (RefSet^XLMSR^) contained more than 80% of proteins at identical frequencies. **(E)** Schematic illustrating the assembly of the PDB reference set (RefSet^PDB^) and filtering steps. The final PDB reference set only includes C+ proteins that had DockQ scores (from comparing predictions to experimental structures) > 0.23. **(F)** Schematic illustrating how pairs from the RefSets were partitioned into different subsets for use as either training datasets to build classifiers or as testing datasets to evaluated classifier performance post-training. See Table S6 for all pairs used during training and testing.

We first constructed the negative set. Previous estimates suggest that the human interactome might contain up to ~1.5 million interactions^38–41^, implying that >99% of the ~200 million possible protein pairs do not interact (are negative). Therefore, to generate a set in which the vast majority of pairs are negative, we folded 40,000 random, binary pairs with our standard folding pipeline. Of these, 9,957 (~25%) were C+ (Figure 2A). To discriminate against pairs that are in the same pathway or complex but do not interact directly, we also compiled a negative set of 3,770 “decoy” pairs that reside in well-characterized multi-subunit complexes such as the proteasome but do not directly interact. 24% (905) of these were C+ (Figure 2B).

We also sought to create a positive reference set that reflects undiscovered interactions that a screen is designed to uncover. Using only structures from the PDB might bias training towards protein complexes that are of current interest and amenable to structure determination. We therefore mined cross-linking mass spectrometry (XLMS) datasets, which capture PPIs in their physiological setting^42^. While this type of data is also biased towards abundant proteins, the residue proximity information provided by the crosslinks and the overall diversity of interactions sampled made it an attractive training set. We compiled 8,685 unique binary human protein pairs based on cross-linked peptides from 20 XLMS studies (Table S2). These were folded using AF-M, which yielded 3,226 C+ positive pairs, from which we selected only pairs in which at least one of the cross-links reported agreed with at least one of the three AF-M models (cross-linked residues located within 36 Å of each other, the upper bound of cross-linker lengths used). After applying these criteria, 1,597 XLMS pairs remained (Figure 2C).

We also sought to create a negative dataset that mirrored the XLMS dataset as closely as possible. To this end, we randomly paired all unique proteins in the XLMS data (after homology reduction, as described below) and folded them via AF-M (Figure 2D; “XLMSR”). This process was repeated iteratively until XLMS and XLMSR sets contained more than 80% of the same proteins at identical frequencies (Figure S2D).

The XLMS set probably contains some false positives. Therefore, to generate a ground truth for independent evaluation of metrics, we also folded protein pairs corresponding to structures in the PDB that were deposited after the AF-M v2.3 training cutoff. This set of 1,288 heterodimeric pairs was folded with templates disabled to avoid accessing PDB information deposited after training. Of these, 946 yielded C+ AF-M predictions, which were compared to the corresponding PDB structures using DockQ^43^. 541 (57.2%) had DockQ > 0.23, the CAPRI cutoff for acceptable model quality (Figure 2E).

After assembling these five datasets, we ensured that no pair displayed 30% or more sequence identity to both partners in any other pair. This avoided any overlap or “data leakage” between training and test sets. Furthermore, to reduce false negatives in training, we purged all negative training sets of pairs with >30% sequence similarity to any interacting pair in the PDB. These homology reduction steps yielded two final positive sets, RefSet^XLMS^ (n = 1,221) and RefSet^PDB^ (n = 410), and three negative sets, RefSet^Random^ (n = 9,852), RefSet^Decoy^ (n = 688), and RefSet^XLMSR^ (n = 1,103). Pairs from these RefSets were assigned to either training or testing datasets, as outlined in Figure 2F.

We first used these curated datasets to further evaluate canonical AF-M metrics. As shown in Figure S2E, 3.3%,19.4%, and 13.5% of the negative pairs (RefSet^Random^ + RefSet^Decoy^) exhibited “positive” pDockQ2, ipTM, and avg_models scores, respectively, even though at most ~0.75% of these random and decoy pairs should be positive^38^. In contrast, the RefSet^PDB^ had many more positive hits, as expected (Figure S2E). However, for all three metrics, there was significant overlap between positive and negative pair distributions (Figure S2E). Together, these results are consistent with the poor performance of these metrics in ranking experiments (Figure 1B–C and Figure S2B–C), underscoring the need for a better metric that distinguishes positive and negative predictions.

We also asked how existing metrics stratify the XLMS dataset that would be used for training (next section). As expected, pairs from RefSet^XLMS^ displayed higher mean pDockQ2, ipTM, and avg_models scores than RefSet^Random^ + RefSet^Decoy^ + RefSet^XLMSR^ (Figure S2F). Notably, a substantial number of RefSet^XLMS^ pairs exhibited low pDockQ2, ipTM, and avg_models scores. It is presently unclear whether this was because RefSet^XLMS^ contains a substantial number of negative pairs, or because it contains true interactions that are difficult to detect using conventional metrics.

### A classifier that distinguishes positive and negative AF-M PPI predictions

To improve upon standard metrics in assessing AF-M predictions, we trained a random forest machine learning model^44^ on the curated datasets. Random forests use random feature subsets during training to build decision trees that each attempt to assign the correct class to each instance of training data (Figure S3A). The resulting algorithm is called a classifier. During inference, previously unseen instances are voted on by all trees, yielding a single classifier score that ranges from 0 to 1. We initially trained only on RefSet^XLMS^ and its randomized counterpart RefSet^XLMSR^, in which protein pairs are closely matched. To train a “structural classifier” on these sets, we extracted several numeric features from the predicted interface of each pair (Table S3 for definitions). These included not only AF-M-based metrics (PAE, pLDDT, and avg_models scores), but other measurable properties of the interface such as the number of salt bridges and hydrogen bonds between interacting residues. We then used iterative pruning to remove uninformative features. The resulting classifier assigned high scores to a large percentage of negative pairs (RefSet^Random^ and RefSet^Decoy^) (Figure S3B). We therefore retrained the structural classifier by also including a fraction of RefSet^Random^ (Figure S3C) or RefSet^Random^ and RefSet^Decoy^ (Figure S3D). This reduced the scores for false positives (Figure S3C), which is desirable given AF-M’s propensity to generate such predictions in larger numbers.

**Figure 3:**
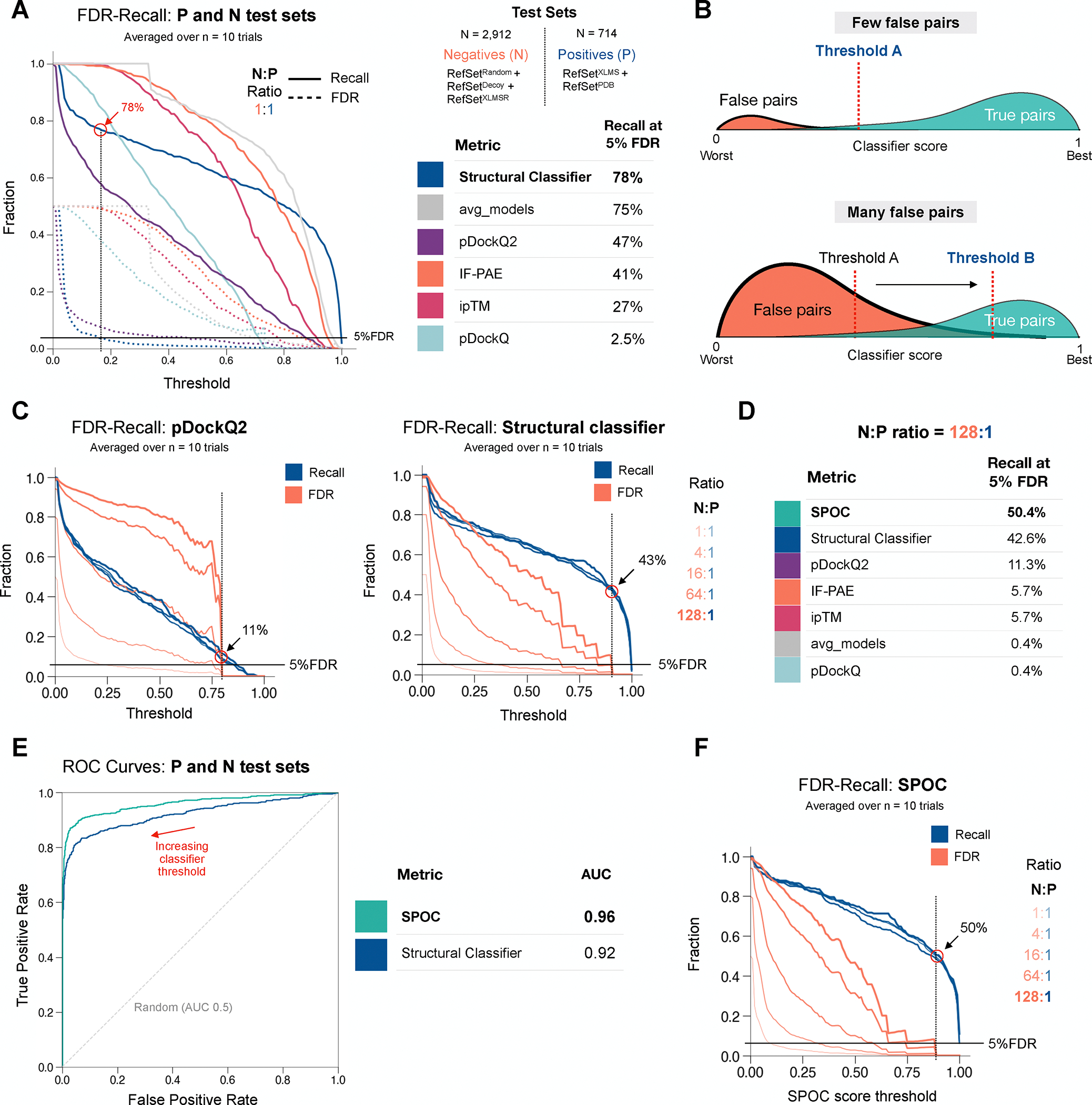
Classifier performance on curated test sets **(A)** A plot of Recall (solid lines) and FDR (dotted lines; same as 1 - precision) as a function of selected threshold for five previously used metrics and the structural classifier at a N:P ratio of 1:1. N is (RefSet^Random^ + RefSet^XLMSR^ + RefSet^Decoy^); P set is RefSet^XLMS^+ RefSet^PDB^. **(B)** Schematic illustrating that as the abundance of false pairs rises, a higher classifier threshold is required to maintain a low FDR. **(C)** Recall-FDR plots for pDockQ2 and the structural classifier at various N:P ratios. N is (RefSet^Random^ + RefSet^XLMSR^ + RefSet^Decoy^); P set is RefSet^XLMS^ + RefSet^PDB^. **(D)** Table comparing the recall fraction at 5% FDR and N:P = 128:1 for pDockQ2 (panel C), structural classifier (panel C), and other metrics (curves not shown). **(E)** AUC under the Receiver Operating Characteristic (ROC) curves for SPOC and the structural classifier. N is (RefSet^Random^ + RefSet^XLMSR^ + RefSet^Decoy^); P set is RefSet^XLMS^+ RefSet^PDB^. **(F)** Recall-FDR plots for SPOC at various N:P ratios. N is (RefSet^Random^+ RefSet^XLMSR^ + RefSet^Decoy^); P set is RefSet^XLMS^+ RefSet^PDB^.

After training the structural classifier on 75% of the data (RefSet^XLMS^, RefSet^PDB^, RefSet^XLMSR^, RefSet^Random^, and RefSet^Decoy^), we evaluated its performance on the 25% testing data held back. Performance was assessed by how many positives (RefSet^XLMS^, RefSet^PDB^) and negatives (RefSet^XLMSR^, RefSet^Random^, and RefSet^Decoy^) were identified above each classifier threshold (“True Positives”, TPs and “True Negatives”, TNs, respectively). Results were displayed as the Recall rate (Figure 3A, solid lines; fraction of all TPs captured above the threshold) and FDR (Figure 3A, dotted lines; fraction of pairs captured above the threshold that are TNs). As a single measure of performance, we determined the Recall rate when the FDR was 5% (1 in 20 interactions labeled by the classifier as true are false). Compared to existing metrics, the structural classifier exhibited the highest recall (78%) at 5% FDR (Figure 3A; table). We also evaluated performance using Receiver Operating Characteristic (ROC) curves (Figure S4A), which quantify the TP and false positive (FP) rates as a function of classifier score. Using this analysis, the structural classifier and avg_models performed comparably, achieving Area Under the ROC Curve (AUC) values of 0.92 and 0.91 respectively. Of note, recall rates and derived statistics were calculated only for pairs that met the C+ interaction criteria; false negatives where AF-M did not predict contact were not considered. Therefore, estimating the absolute recall rate requires normalizing by the AF-M true positivity rate (40%), which is derived from the fraction of the 1,288 PDB RefSet pairs with an AF-M prediction achieving a DockQ score > 0.23 (Figure 2E).

As in many other studies, the above evaluation of classifier performance involved test sets with equal numbers of negatives and positives (reviewed in ^45^). However, this 1:1 N:P ratio does not accurately reflect most real-world scenarios, the most extreme of which occurs when a single protein is screened against the entire proteome. In this case, we estimated the N:P ratio of contact positive AF-M predictions to be ~80:1 (Figure S3E). In general, as the N:P ratio increases, so does the number of negative pairs with high scores (false positives); due to this “invasion” by negatives, the threshold score must be increased to maintain an acceptably low FDR (Figure 3B). Therefore, to obtain a more realistic estimate of classifier performance, we created test sets spanning N:P ratios from 1:1 to 128:1. As expected, the Recall of positives was unaffected by these ratios, but the FDR increased progressively as the proportion of negatives increased (Figure 3C). At the 128:1 ratio and 5% FDR, the structural classifier retained the highest recall (42.6%), greatly outperforming the next closest metric pDockQ2 (11.3%) (Figure 3C–D; see Figure S4B for standard precision recall curves). Therefore, the structural classifier outperformed other metrics under conditions mimicking a proteome-wide screen.

We wondered whether classifier performance could be boosted further by considering biological properties of interacting proteins. We therefore included genome-wide features of each protein pair that were external to AlphaFold, including co-dependency data from DEPMAP^46^, coexpression data^47^, T5 protein language model embeddings^48^, subcellular colocalization predictions from DeepLoc 2.0^49^, hit profiles from the BioORCS CRISPR screen database, and high throughput interaction experiments from the BioGRID database^50^ (Table S3). All features were derived by combining the metrics of both proteins so that the classifier could not identify or learn from protein identities within pairs. The resulting Structure Prediction and Omics informed Classifier (SPOC) outperformed all other indicators, including the structural classifier: SPOC’s AUC in ROC curves was 0.96 (Figure 3E), and at the 128:1 N:P ratio, it recalled 50.4% of positive pairs at 5% FDR (Figure 3D and 3F). Strikingly, above a score of 0.89, SPOC achieved an effective FDR of 0% on the test sets while still recalling ~50% of C+ positives, even at the highest N:P ratio (Figure 3F). Correcting this recall rate by the ~40% true positive rate of our protocol (Figure 2E) produces an estimated absolute recall rate of 20% for SPOC on all PPIs.

As expected from SPOC’s superior performance over the structural classifier, it is driven by both ‘biological’ and ‘structural’ features. Based on their GINI importance scores, structural and biological features collectively contributed ~55% and ~45% to performance, respectively (Figure S4C). Among structural features, a metric quantifying the minimum number of contacts across all models was most important (12%), and among biological features, it was the number of independent experiments supporting an interaction in the BioGRID database (21%). Although some BioGRID data is derived from studies focusing on a specific process or protein, the vast majority involves unbiased interaction evidence mined from proteome-wide screening efforts. Our results suggest that, by evaluating structural and biological features of protein pairs, SPOC identifies true interactions in a curated dataset with good sensitivity and high specificity, even at N:P ratios that approximate proteome-wide screens.

### SPOC enables proteome-wide screening for PPIs

**Figure 4:**
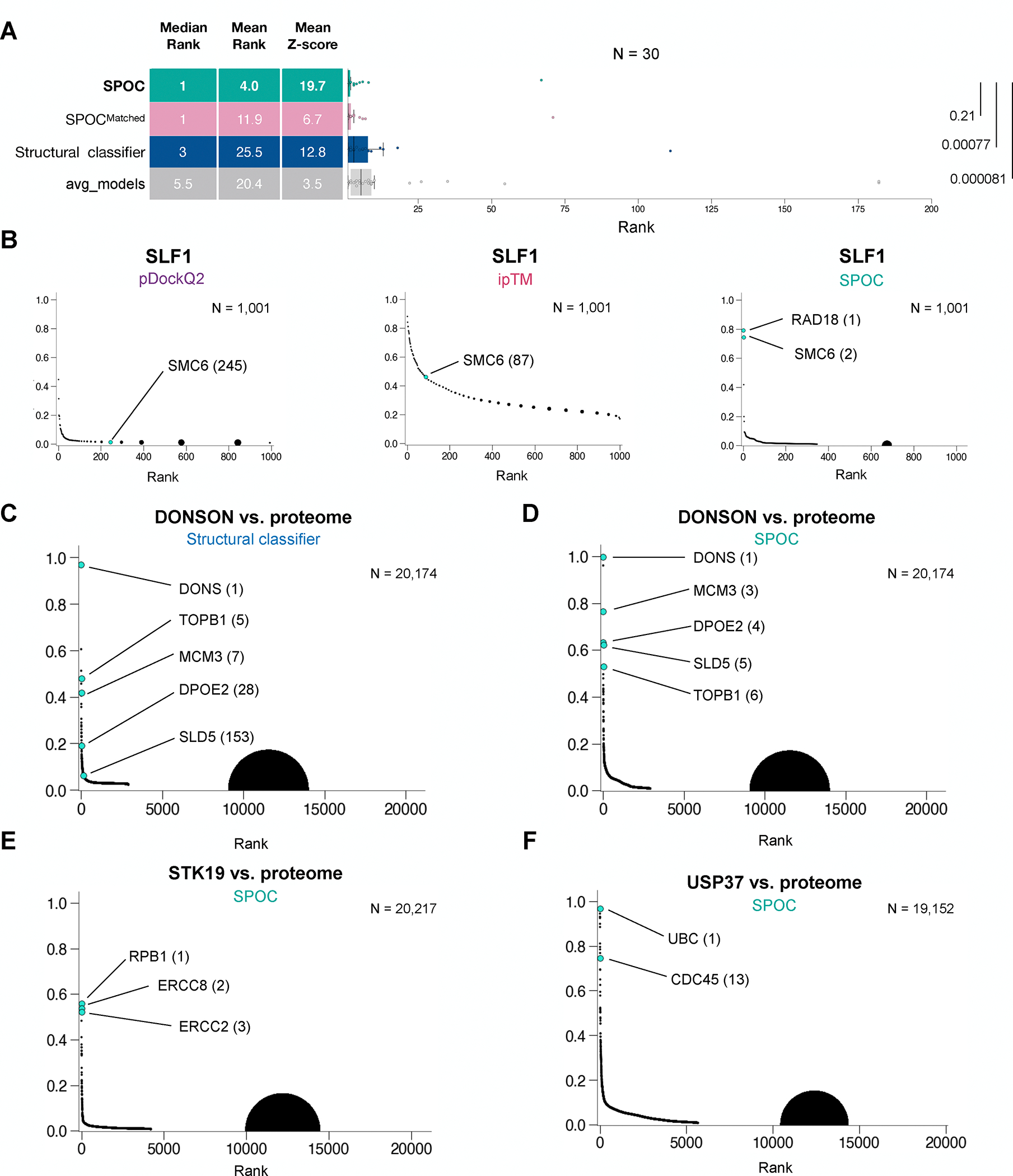
Evaluating SPOC performance for biological discovery applications **(A)** Box plots comparing the performance of four ranking metrics. The data for avg_models are the same as in Figure 1C. X-axis was truncated above rank 200. P-values shown are from two sided Wilcoxon signed-rank test of all metrics compared to SPOC. **(B)** The SLF1/SMC6 true pair was embedded in 1000 random pairs involving SLF1, and the AF-M predictions for each pair were ranked using three different metrics. **(C)** DONSON was folded with more than 20,000 human proteins, and the resulting predictions were ranked using the structural classifier. True DONSON interactors are indicated in cyan. **(D)** Same as (C) but using SPOC for ranking. **(E)** STK19, a recently identified TC-NER factor, was folded against more than 20,000 human proteins and the predictions were ranked by SPOC. Verified interactions are shown in cyan. **(F)** USP37, a de-ubiquitinating enzyme associated with the replisome, was folded against more than 19,000 human proteins and the predictions were ranked by SPOC. Verified interactions are shown in cyan.

To assess SPOC in real-world scenarios, we revisited the ranking tests (Figure 1A), an orthogonal measure of performance compared to classification of curated datasets. SPOC outperformed all other metrics in these ranking experiments (median rank = 1, mean rank = 4)(Figure 4A–B) (Table S1). Importantly, SPOC performed well on proteins in different compartments and pathways, as expected given that training was pathway- and compartment-agnostic (Table S1). Moreover, whereas conventional metrics generally distributed pairs evenly across their respective score ranges, SPOC more clearly separated the TP from TNs (Figure 4B, Figure S5B–D). This separation of true pairs from background is quantified by the Z-score, which is highest for SPOC (Figure 4A). Notably, in addition to the desired prey SMC6, another top ranked hit for SLF1 was RAD18, a known SLF1 interactor^51–53^ that was randomly included as part of the 1,000 protein “negative” set. We also assessed how SPOC ranked DONSON’s five interactors (MCM3, SLD5, TOPB1, DPOE2, and DONSON; Figure S2A) in our proteome-wide screen for DONSON interactors^23^. Unlike all other metrics (Figure S2B–C) including the structural classifier (Figure 4C), SPOC placed DONSON’s functional partners in the top 6 hits out of more than 20,000 pairs (Figure 4D). This was remarkable given that neither DONSON nor any of its homologs were present in any training data sets. We also performed a proteome wide interaction screen for STK19, which we recently showed is essential for transcription-coupled nucleotide excision repair ^54^. SPOC ranked its functional interactors (RPB1, ERCC2, and ERCC8)^55^ in the top three hits (Figure 4E). Finally, a proteome wide screen for USP37 interactors ranked many ubiquitin-related proteins highly, as expected, as well as CDC45, which we recently implicated in tethering USP37 to the replisome (Figure 4F)^56^. Consistent with our data above (Figure S3B–D), training on a matched, but less complex negative set modestly reduced SPOC’s performance (Figure 4A, SPOC vs. SPOC^Matched^, compare mean ranks, and Figure S5A). Collectively, our results show that SPOC outperforms all other metrics in real-world ranking experiments and can help discover PPIs *ab initio* in proteome-wide *in silico* screens.

### *In silico* screening in genome maintenance

**Figure 5:**
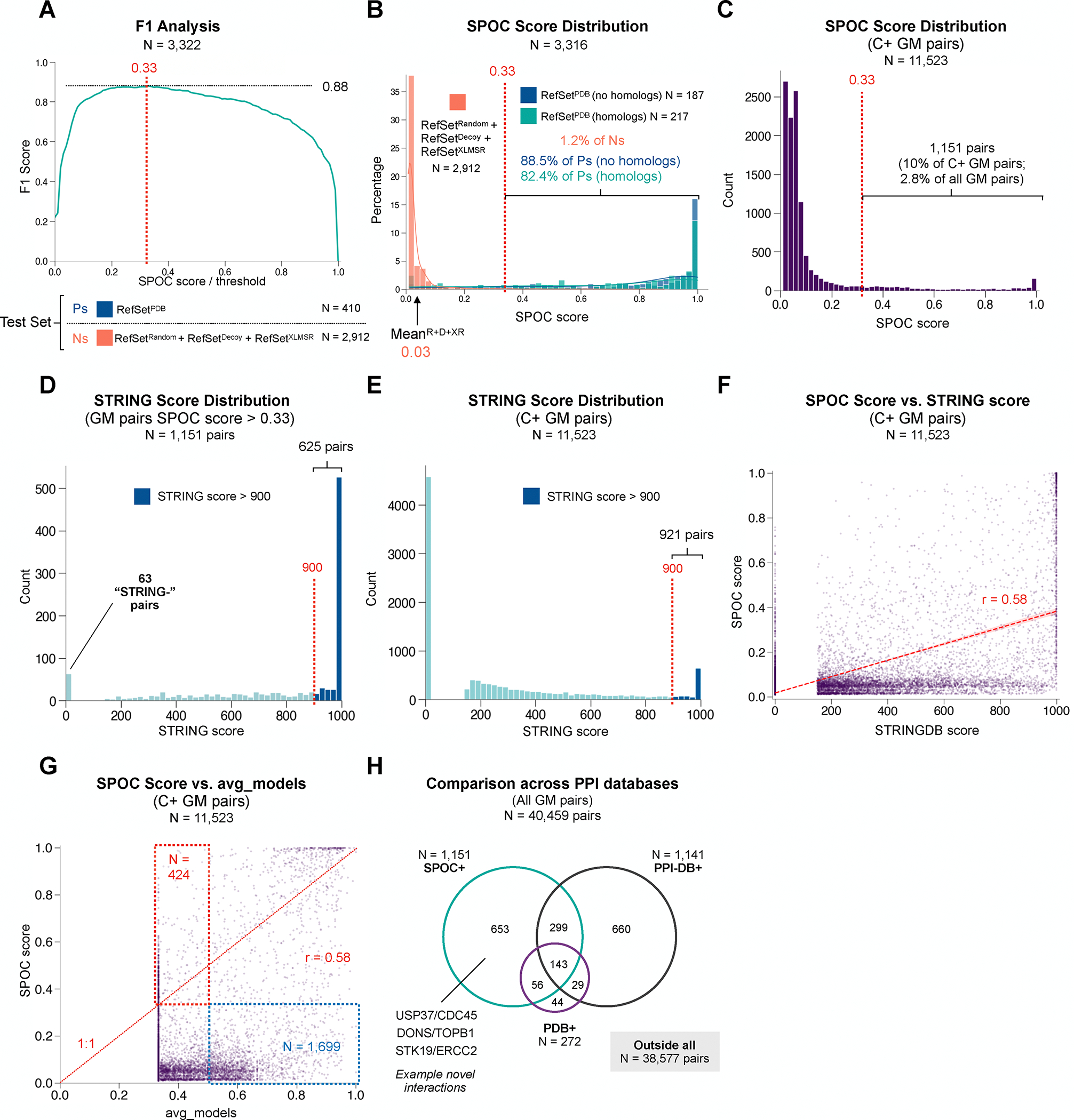
Applying SPOC to find interactions in a biological pathway **(A)** A plot of the F1 score (TP/(TP + 0.5*(FP + FN)) as a function of SPOC score/threshold applied to the N (RefSet^Random^ + RefSet^XLMSR^ + RefSet^Decoy^) and P (RefSet^PDB^) test sets. The F1 score peaks at a threshold SPOC score of 0.33. **(B)** Histogram showing the distribution of SPOC scores for TNs (RefSet^Random^ + RefSet^XLMSR^ + RefSet^Decoy^) and TPs (RefSet^PDB^) split into pairs with and without homologs in the PDB prior to the AF-M v2.3 training cutoff. A proposed cutoff for true interactions (0.33), as well as the % of TPs and TNs above the cutoff are shown. **(C)** The distribution of SPOC scores on 11,523 C+ Genome Maintenance (GM) pairs. 10% of these interactions achieved a SPOC score > 0.33. **(D)** Histogram of STRING DB scores associated with the 1,151 pairs with SPOC score > 0.33 shows that 625 (54.3%) top classifier scoring interactions also have high (>900) STRING scores. **(E)** Histogram of STRING DB scores for all 11,523 C+ GM pairs. 4,582 pairs have scores of 0, indicating that no prior text or data has suggested a potential association while 921 (8%) have scores greater than 900. **(F)** A scatter plot comparing the SPOC score (y) to the STRINGDB score (x) for the 11,523 C+ pairs in the GM dataset with a dashed red line indicating the best fit line. **(G)** A scatter plot comparing the SPOC score (y) to the avg_models score (x) for the 11,523 C+ pairs in the GM dataset. The dashed red line shows how a perfect equivalence between the two scoring schemes would look. See text for explanation of red and blue boxes. **(H)** A Venn diagram showing how pairs from the GM dataset are distributed across 3 different subsets PDB+, SPOC+, PPI-DB+. See Table S4 for all data relating to GM pairs.

Having developed SPOC, we used it to score all possible pairwise interactions within a biological pathway. We folded nearly all binary combinations of 286 core human genome maintenance (GM) proteins, yielding 40,459 structure predictions. Of these, 11,523 (28.5%) satisfied our contact criteria. To find a suitable SPOC score cutoff that balances recall with precision, we plotted the F1 score (the harmonic mean of precision and recall) as a function of SPOC. This value peaked at a SPOC score of 0.33 (Figure 5A). This score cutoff captured ~89% of RefSet^PDB^ pairs with no homology to complexes AF-M was trained on, ~82% of pairs in the RefSet^PDB^ with homology to AF-M training complexes, and only 1.2% of TNs from RefSet^Random^ + RefSet^XLMSR^ + RefSet^Decoy^ (Figure 5B). Across all GM pairs, 1,151 (2.8%) had SPOC scores > 0.33 (Figure 5C), implying that the average GM protein might have four true partners in the matrix. Among this set, 303 pairs achieve SPOC scores > 0.89, the threshold associated with an FDR of 0 during testing at N:P ratios of 128:1.

We addressed how SPOC compares to curation by STRING, in which association strength scores exceeding 900 (on a 0–1000 scale) indicate strong potential for a physical or functional interaction, and scores below 400 are considered low confidence^57^. Notably, 625 (54.3%) of the 1,151 GM pairs with a SPOC score >0.33 had STRING scores >900 (Figure 5D), compared to 921 among all 11,523 C+ pairs (8%) in the GM group (Figure 5E), a ~7-fold enrichment. 63 of the 1,151 pairs with high SPOC scores (~5%) were absent from STRING and 178 (~15%) had scores below 400 that are considered insignificant (Figure 5D; Table S4). These findings align with the fact that while there is some correlation (r = 0.58) between SPOC and STRING scores, they often diverge (Figure 5F). Among the pairs that score highest by SPOC but are absent from STRING is MMS22L-RPA2, which is consistent with biochemical evidence that the MMS22L-TONSL complex binds to RPA-ssDNA filaments^58^. Another example is USP37-CDC45, which is consistent with our finding that CDC45 recruits USP37 to the replisome^56^. We also addressed how SPOC and the best prior metric in ranking experiments, avg_models, compare in the analysis of the GM data. Plotting the SPOC score vs. avg_models showed some correlation (r = 0.58), but overall low agreement (Figure 5G). Importantly, there were many pairs with low avg_models scores (<=0.5) that SPOC rated highly (>0.33; red box) and conversely, many pairs with high avg_models scores (>0.5) that SPOC downgraded (<=0.33; blue box). These results underscore that SPOC makes substantial adjustments to previous rankings. We also compared our SPOC results with large PPI databases to estimate what percentage of high confidence experimental PPIs (PPI-DB+) are captured via our approach (Figure 5H). This analysis revealed that out of the 40,459 pairs in the GM set, 1,141 are reported in both BioGrid and IntAct^59^. Among these 1,141 pairs, 442 (38.7%) had SPOC scores > 0.33 while the remaining ~60% were assigned scores below the SPOC threshold. Many of these almost certainly represent indirect interactions captured in PPI databases.

The GM group contains many PPIs with high SPOC scores that are not structurally resolved but are nevertheless supported by strong biochemical or genetic evidence, indicative of SPOC’s ability to detect meaningful interactions. In several instances, the PPI has also been mapped sufficiently to indicate that the structure prediction is likely correct. An example is the CIP2A-TOPBP1 pair (SPOC=0.898), in which residues shown to be critical for the interaction^60^ agree with the AF-M prediction (see predictomes.org; Note: revised SPOC scores based on retraining for this revision will be updated at predictomes.org). Other examples include CIP2A-CIP2A (SPOC=0.998; ^61^), FIGNL1-FIRRM (SPOC=0.988;^62,63^), and MMS22L-TONSL (SPOC=0.993;^64–66^)(Table S4). This “retrospective validation” suggests that the GM dataset contains many valid predictions, and that *in silico* screening is a powerful approach to discover PPIs.

### A web portal for AlphaFold multimer predictions

**Figure 6:**
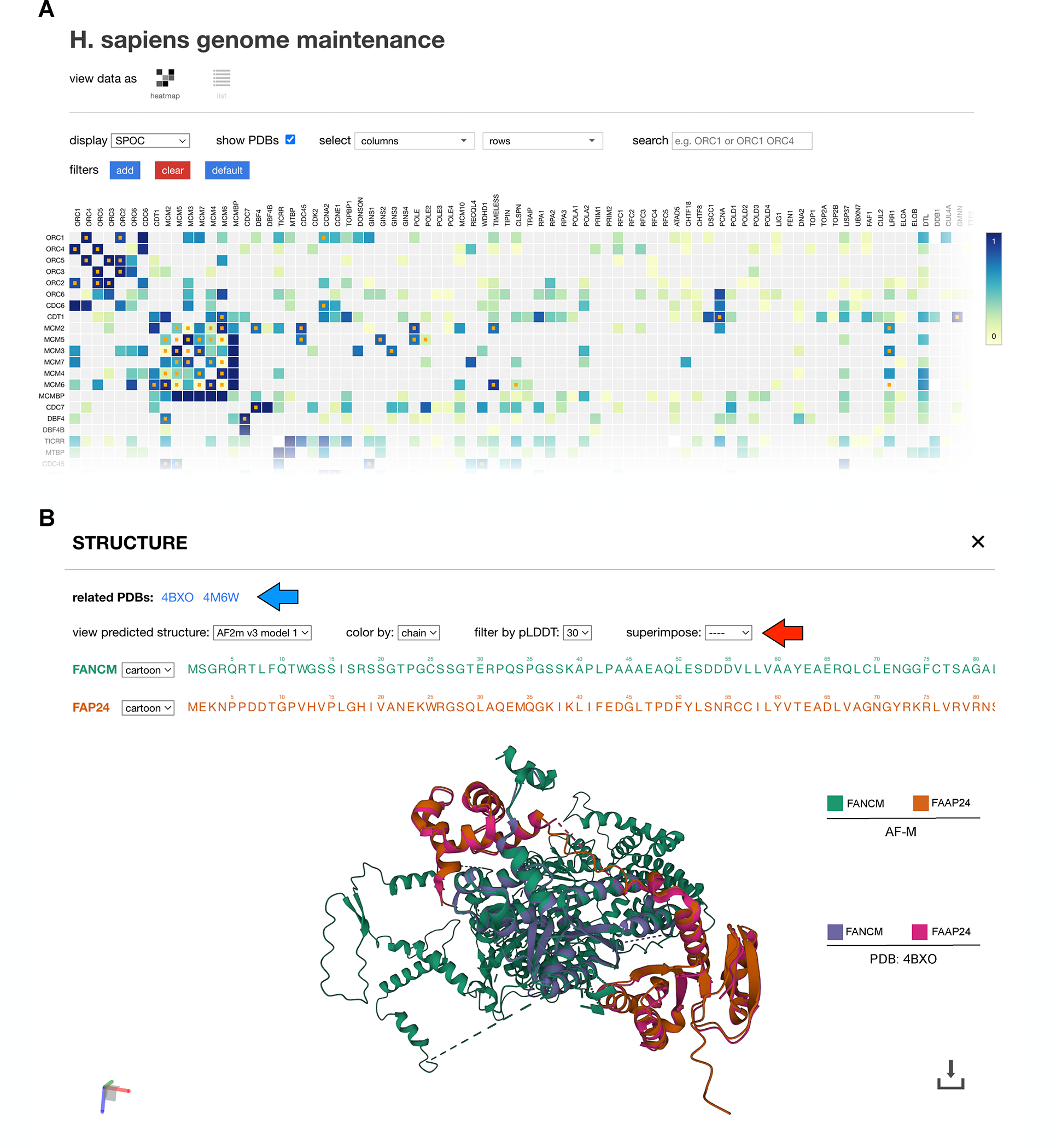
A web portal for AF-M predictions **(A)** Screenshot of the interactive matrix from predictomes.org. Tile color darkness is proportional to the displayed confidence metric (SPOC). Tiles with orange dots represent pairs that are found in the PDB. Specific biological pathways can be selected for display in rows and columns. **(B)** Screen shot of the interactive structure viewer for the FAAP24-FANCM pair. The FANCM-FAAP24 structure (PDB 4BXO; purple and pink chains) was superimposed on the AF-M structure prediction (green and orange chains) using the superimpose tool (red arrow). There are different options to display the structure, filter residues by pLDDT, and color the structures by different metrics such as pLDDT.

To allow researchers to interact with the GM data, we created predictomes.org, a user-friendly online database. Users can browse an interactive matrix (Figure 6A) or a sortable list (Figure S6A) that can be ranked by SPOC score and other metrics. Clicking on a matrix tile or a list entry displays an information page that includes an interactive protein structure viewer (Figure 6B)^67^ from where the structure predictions can be downloaded. If experimental structures of the pair already exist, the corresponding PDB entries are listed above the structure viewer (Figure 6B; blue arrow), and the PDB structures can be superimposed on the AF-M prediction (Figure 6B; red arrow). The information page also contains UniProt entry information, residue level evolutionary conservation, predicted residue contacts, interactive PAE and pLDDT plots, and data from the STRING and BioGRID databases about potential associations (Figure S6B and predictomes.org). These features allow rapid visualization, ranking, and triage of thousands of structure predictions.

### AI-driven hypothesis generation

**Figure 7:**
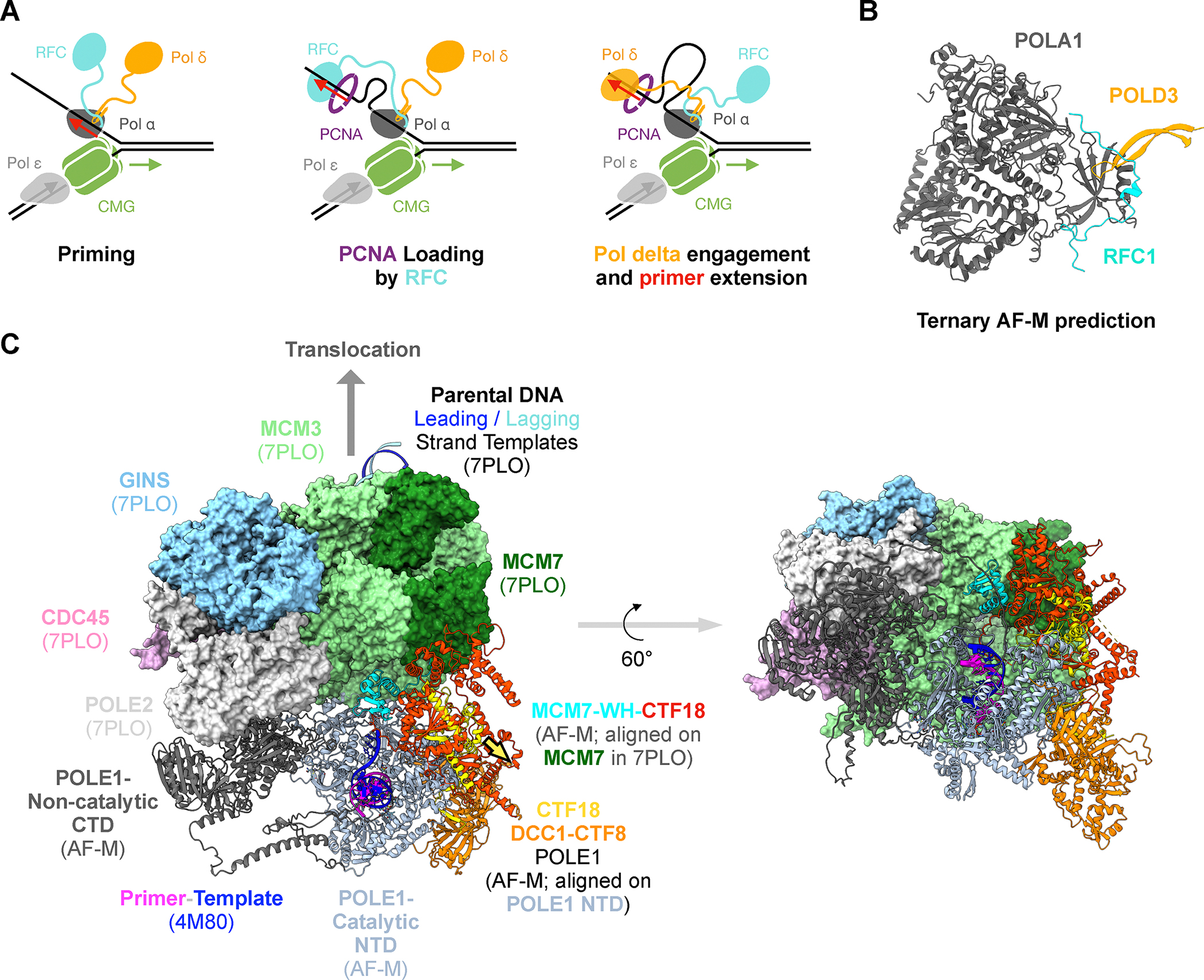
Hypotheses suggested by predictomes.org **(A)** Model of processive Okazaki fragment processing. During Okazaki fragment priming by pol α, pol δ (via POLD3) and RFC (via RFC1) bind cooperatively to pol α (via POLA1). As soon as pol α releases the primer, the tethered RFC occupies the primer-template and deposits PCNA. When RFC dissociates, the tethered pol δ occupies the primer-template and initiates primer extension. **(B)** AF-M prediction of POLA1 (grey; residues 310–1264), POLD3 (yellow; residues 380–412), and RFC1 (cyan; residues 153–190) folded at the same time. For POLA1-POLD3 and POLA1-RFC1 binary predictions, see figures S7A–B and predictomes.org. **(C)** AF-M-informed model of how the POLE1 catalytic domain is positioned near CMG’s exit channel by the interaction of CTF18 (red) with the winged-helix domain of MCM7 (cyan) and the POLE1 catalytic domain. The model was assembled as follows: An AF-M prediction of POLE1 (C-terminal non-catalytic domain shown as grey ribbon, N-terminal catalytic domain shown as light blue ribbon) was aligned on the C-terminal, non-catalytic lobe of POLE1 in the cryo-EM structure of the human replisome (PDB: 7PLO^78^, of which only MCM2–7, CDC45, and GINS are shown, POLE1 hidden). To model the primer template, the structure of yeast POLE1 catalytic domain with a primer template (PDB: 4M8O) was aligned on the POLE1-NTD shown (yeast POLE1 hidden; primer-template shown). We also generated an AF-M prediction of a complex of CTF18, CTF8, DCC1, and the NTD of POLE1 (which matches key features of an analogous experimental structure, PDB 6S2E;73), and aligned it on the POLE1-NTD shown. This revealed that the CTF18 (yellow)-DCC1(orange)-CTF8(orange) complex binds the distal side of POLE1-NTD. Separately, MCM3, MCM7, and CTF18 were folded together and aligned on MCM7 from 7PLO. This shows that a movement of 30Å (yellow arrow) would superimpose the CTF18 aligned on MCM7 (red) and the CTF18 aligned on POLE1-NTD (yellow). Given the reported flexibility between the NTD and CTD of POLE174, and some predicted flexibility between the NTD and CTD of CTF18 (https://alphafold.ebi.ac.uk/entry/Q8WVB6), CTF18 should be able to bind MCM7 and POLE1-NTD simultaneously. In this way, CTF18 would tether the POLE1-NTD near the rear exit channel of CMG, with the leading strand template (dark blue strand) being fed into the active site.

The genome maintenance predictome contains many high confidence predictions that suggest interesting and testable hypotheses. We highlight two examples related to replicative DNA polymerases. The first involves lagging strand synthesis (Figure 7A). In this process, DNA polymerase α (Pol α), which interacts stably with the CMG replicative helicase^68^, first primes each new Okazaki fragment on the lagging strand template. RFC then loads the processivity factor PCNA on these primers, followed by primer extension by Pol δ. Interestingly, AF-M predicted with high confidence (SPOC=0.947) that the non-catalytic POLD3 subunit of Pol δ extends an exposed beta sheet in the catalytic POLA1 subunit of Pol α (Figure 7B and S7A; Table S5 for other confidence metrics). This interaction was predicted from humans to fission and budding yeasts (Table S5), and in budding yeast, previous experiments mapped this interaction to the location in both proteins predicted by AF-M^69,70^. Interestingly, the same region of POLA1 was also predicted to bind a small peptide in the N-terminal unstructured region of RFC1 (SPOC=0.743), the largest subunit of the RFC complex (Figure 7B and Figure S7B; Table S5). Strikingly, in three-way structure predictions, the POLD3 and RFC1 peptides interacted with each other on the surface of POLA1, with RFC1 draping over the composite beta sheet formed by POLA1 and POLD3 (Figure 7B). This ternary complex was predicted with high confidence across metazoans and fission yeast but not in budding yeast (Table S5; SPOC currently only scores binary predictions), and it is consistent with isolation of a Pol δ - Pol α -RFC complex from mammalian cells^71^. These predictions suggest that when Pol α primes a new Okazaki fragment, RFC and Pol δ are already attached to Pol α, allowing seamless transfer of the primer from Pol α to RFC to load PCNA, followed by engagement of Pol δ and primer extension (Figure 7A). In agreement with this model, single molecule experiments demonstrate that yeast Pol δ remains bound to the replisome over multiple cycles of Okazaki fragment synthesis, an effect that depends on Pol32, the yeast counterpart of POLD3^72^.

The second hypothesis we highlight addresses how the leading strand DNA polymerase ε (Pol ε) and the CTF18 complex are oriented on the replisome. In particular, the location of the catalytic domain of Pol ε on the replisome remains mysterious. AF-M predicted an interaction between CTF18 and the winged helix domain of MCM7 (SPOC = 0.466), which resides near the rear exit channel of CMG (Figure 7C, red and cyan ribbon diagrams; Table S5). Together with the extensive interface between the CTF18 complex and POLE1^73^, these interactions would position the catalytic, N-terminal domain of Pol ε adjacent to CMG’s rear exit channel (Figure 7C, grey ribbon diagram; see figure legend). In this way, the leading strand template (Figure 7C, blue strand) would be fed directly into the Pol ε active site. The above examples illustrate how large-scale screening for binary PPIs leads to mechanistic hypotheses, some of which are readily aligned with existing data.

### SPOC Tool

To facilitate broad access to SPOC, we created an online tool, accessible at predictomes.org, that analyzes user-generated AF-M structures. After uploading their predictions, users are sent SPOC, ipTM, pDockQ, and avg_models scores for each protein pair. We expect that SPOC will facilitate identification of biologically plausible binary structure predictions.

## Discussion

Here, we report tools and resources that will help biologists leverage the structure prediction revolution for mechanistic discovery. First, we generated well-curated sets of positive and negative protein pairs that can direct future machine learning efforts. Second, we used these datasets to train SPOC, a classifier that effectively discriminates biologically meaningful and false positive AF-M predictions. The power of SPOC is illustrated by the fact that in proteome wide screens, it readily identified STK19, USP37, and DONSON partners whose function we validated^23,54,56^. We make SPOC available online to allow classification of user-generated structure-predictions. Third, we present a SPOC-curated structural predictome of genome maintenance proteins, and we give examples of how it powers hypothesis generation. Together, the work helps lay the foundation for the eventual development of a comprehensive structural interactome.

Two independent forms of evidence show that SPOC outperforms all previous metrics. First, on curated testing sets containing positive and negative pairs, SPOC exhibits the highest AUC values in ROC curves (0.96 vs. next best of 0.92), and it captures the largest number of positive interactions under realistic screening scenarios where negatives greatly outnumber positives (50% vs. 11% for pDockQ2, the next best-performing conventional metric). Second, in orthogonal performance tests involving *in silico* screens, including three that were proteome-wide, SPOC ranked positive pairs higher than any other metric. We discovered that a classifier trained on well-matched positive and negative datasets (SPOC^Matched^) is slightly inferior to SPOC trained on a dataset that better reflects the high ratio of negative to positive pairs seen in cells. We infer that SPOC outperforms SPOC^Matched^ because the former was exposed to a more diverse set of negative examples rather than data leakage between training and testing sets. Indeed, protein identities within pairs were obscured in the pairwise features used during training, and the ranking experiments demonstrate that SPOC performed better than SPOC^Matched^ in real-world tests that are unrelated to training. This correspondence between RefSet testing performance and orthogonal real-world tests suggests that SPOC has learned meaningful decision boundaries rather than flawed strategies derived from data leakage during training and testing.

An important consideration is how to use SPOC to identify positive pairs while minimizing false positives. As shown in Figure 5B, positive pairs can in principle have any SPOC score, but they are strongly enriched for high scores. Therefore, the appropriate SPOC threshold depends on context and what FDR is tolerable, which can be judged from the Recall-FDR curves in Figure 3F. A proteome-wide screen is expected to involve an ~80:1 N:P ratio and is therefore best modeled by the 64:1 and 128:1 curves, where maintaining a 5% FDR requires a minimum classifier threshold of ~0.89. In contrast, looking for direct interactors after enriching for partners either via pathways analysis or through experiments (e.g. IP-MS pulldown) is more appropriately evaluated at a lower ratio such as 16:1 or 4:1, where 5% FDR is compatible with thresholds of ~0.5–0.75. In some cases, the use of even lower SPOC thresholds may be justified such as the 0.33 cutoff we employed to analyze the predictomes GM matrix. However, regardless of the specific use case or dataset, the best and safest strategy is always to analyze interactions from highest to lowest SPOC score. Although a SPOC score of >0.89 yielded an apparent FDR=0 in curated test datasets, even at the 128:1 ratio (Figure 3F), it is uncertain whether this holds true for real-world data. In other words, a high score alone never provides proof of an interaction, and conversely, a low classifier score is not proof that an interaction is false. Finally, although SPOC was not explicitly trained to report on interface correctness, model quality is implicit because many of the features that contribute to the score are standard AF-M confidence metrics. Despite the above limitations, we believe that when used judiciously, SPOC provides a powerful tool to prioritize AF-M interactions and drive mechanistic discovery.

It is interesting to consider possible sources of erroneous classifications in AF-M screens. False positives might arise because AF-M was only trained on true positives from the PDB and therefore attempts to find an interaction solution for all pairs. Some false positives in this class might in fact interact if brought together in vitro (“biophysical interactors”) but would not do so due to a lack of co-expression or co-localization. Physical interaction studies on such pairs will be required to distinguish these possibilities. Many false negatives probably involve protein pairs that are scaffolded by other factors and whose interaction is too minimal to be detected by AF-M in binary screens. Other false negatives may arise because we folded each pair in only three models to maximize throughput. Some of these can probably be eliminated by folding pairs in all 5 models, increased sampling^74^, or segmenting proteins^75,76^. SPOC almost certainly also has “blind spots.” These will include PPIs with physical features that are not well represented in the XLMS data on which the classifier was trained, as well pairs that are so distinct from any that AF-M was trained on that they cannot be structurally modeled. SPOC is also expected to give low scores to protein pairs that do not exhibit biological patterns (e.g. co-expression, co-immunoprecipitation, genetic co-dependence) typically associated with interacting proteins. An example would be a PPI in which the two interacting proteins’ primary functions and partners are in orthogonal pathways. Finally, while a pair’s SPOC score will likely suffer if it rates poorly in BioGrid, the score can still be strong based on a pair’s other features (e.g. DONSON/TOPB1, USP37/CDC45, and STK19/ERCC8). To improve SPOC, training on more diverse structural and biological datasets will likely be required.

A web version of SPOC is accessible at predictomes.org and calculates scores for researcher-generated AF-M predictions. This tool works best when applied to predictions generated using AF-M settings that resemble those used to train the classifier. Accordingly, if a pair was folded in all five models, the tool randomly analyzes three to mirror our training regimen. However, other AF-M settings such as the number of recycles or dropout enabling that cannot be adjusted post-hoc may impact predictions. In many cases, it will therefore be advisable to regenerate and analyze AF-M structures using the same protocol used for classifier training.

The data in predictomes.org catalyzes mechanistic discovery. By sorting each protein’s putative partners as a ranked list and displaying predictions in an interactive structure viewer with relevant, accompanying information, users can rapidly triage vast numbers of structure predictions and formulate hypotheses. Even in a well-defined pathway such as genome maintenance, comprehensive *in silico* screening highlights interactions that appear to be “hiding in plain sight,” such as the predicted interaction between POLA1 with POLD3 and POLA1 with RFC1. Such binary predictions often motivate higher order folding experiments involving three or more proteins. For example, AF-M predicted the existence of a ternary complex of pol δ, RFC, and pol α that promotes Okazaki fragment synthesis in a processive assembly line, consistent with single molecule experiments^72^. Generating, organizing, and classifying structure predictions in major biological pathways, and eventually proteome-wide, has the potential to launch a new era of mechanistic discovery in the biological sciences.

### Limitations of the study

Two additional limitations of our analysis are worth noting. First, our analysis currently ignores pairs where AF-M fails to produce an interface prediction and thus will miss any positive interactions not predicted by AF-M. Second, while we have used “retrospective validation” of known PPIs to demonstrate SPOC’s performance, large-scale experimental validation of predicted pairs (e.g. by XLMS) will be required to further assess SPOC’s ability to identify previously unrecognized PPIs.

## Resource Availability

### Lead Contact

Further information and requests should be directed to and will be fulfilled by the lead contact, Johannes Walter (johannes_walter@hms.harvard.edu)

### Materials Availability:

No new laboratory reagents were generated during this study.

### Data and Code Availability:

- All AlphaFold multimer models and data related to this manuscript have been made publicly accessible on the SBGrid data repository: 10.15785/SBGRID/1155. Analysis files associated with the manuscript have similarly been published on Zenodo: 10.5281/zenodo.14641589. Accession numbers for previously published datasets used in this study are listed under the Key Resources Table.
- All code relevant to this study have been published on Zenodo:10.5281/zenodo.14641589. An archived copy of the first version of the GitHub repository for the SPOC command line tool (https://github.com/walterlab-HMS/SPOC) is also available on Zenodo: 10.5281/zenodo.14768322.
- Any additional information required to re-analyze the data reported in this paper is available from the lead contact upon request

## STAR Methods

### METHOD DETAILS

#### AlphaFold-Multimer (AF-M)

We used a locally installed version of ColabFold^77^ v 1.5.2 to run AF-M. All our predictions used AF-M multimer version 3 weights models 1, 2, and 4 with 3 recycles, templates enabled, 1 ensemble, no dropout, and no AMBER relaxation. The Multiple Sequence Alignments (MSAs) supplied to AF-M were generated by the MMSeq2^79^ server using default settings. All MSAs consisted of vertically concatenated paired and un-unpaired MSAs for the query proteins. The majority of predictions were run on 40GB A100 NVIDIA GPUs while a subset was run on L40S NVIDIA GPUs. Given the memory limitations of these GPUs, we generally cap all jobs at 3,600 amino acids total. In certain cases where a structure is of particular interest, we make exceptions and run AF-M on sequences exceeding 3,600 amino acids.

#### Finding contacts in AF-M structures

To determine which residue pairs make valid interfacial contacts in AF-M structures, we use a multi-tiered filtering approach that considers distance along with the pLDDTs and PAE scores. The first step is to iterate through the structure and find all residue pairs in which at least 1 pair of heavy atoms are < 5Å apart and where both residues have pLDDTs > 50. To avoid clashes, we then eliminate pairs with any heavy atoms closer than 1Å. Next, the two PAE scores associated with each residue pair (x, y and y, x) are examined, and if they are < 15 Å, this residue pair is added to the list of valid interfacial contacts. This pipeline differs from our previous approach^23^ where our distance limit was < 8 Å and we did not consider clashes.

#### Genome maintenance pair generation

Based on literature and expert curation we selected 286 human proteins. We then generated all possible unique binary pairs of these proteins and proceeded to fold them in 3 AFM models (models 1,2,4). Due to GPU memory restrictions and time limits, we almost always limit the total amino acid length of pairs we folded to below 3,600 residues. In rare cases where proteins are of particular interest to the community (i.e. BRCA2) we sometimes exceeded this limit and ran pairs that exceeded 3,600 amino acids in length.

#### Plots

All plot visualizations were generated using the Python library matplotlib and seaborne running online in Google Colab Jupyter Notebooks.

#### PDB human pair contact identification

The PDB API^90^ was used to find all cryo-EM and X-ray crystallography structures with overall resolutions < 3.5 Å containing at least two annotated human protein chains. Once all structures with these criteria were retrieved, we iterated through all possible combinations of human chain pairs to find those in contact. Chains were considered to be in contact if they had at least 10 residue pairs with heavy atoms closer than 5 Å. The PDB SIFTS API was used to map PDB chain entries to their corresponding UniProt identifiers. Based on this analysis of the PDB performed on January 10, 2024, there were 8,472 unique human binary protein pairs.

#### PDB pair retrieval and DockQ Calculation

We first identified all human heterodimeric pairs structurally resolved in the PDB after the AlphaFold-multimer version 3 training cutoff date (September 30, 2021). For each of these pairs, we selected the structure and chain-pair that had the most interfacial residue pair in contact, as defined by our contact criteria (heavy atoms < 5Å). The PDB API was then used to download the atomic coordinates corresponding to each of the chains in CIF format using the following URL: https://models.rcsb.org/v1/{pdb_id}/atoms?label_asym_id={chain}&model_nums=1&encoding=cif&copy_all_categories=false&download=false.

Each chain was extracted from the two individual chain CIF files, re-parsed into PDB format, and combined into a new PDB file containing only those two chains. Double-letter PDB chain codes were remapped to single-letter chain codes to maintain compatibility with the PDB format. We then used the DockQ script from the GitHub repository (https://github.com/bjornwallner/DockQ/tree/v1.0) to measure DockQ scores between the two-chain PDB structure file and each of the three AlphaFold-Multimer (AF-M) predictions (models 1, 2, and 4) made for each pair.

Globally optimal sequence alignments between the native and model chains were performed using the EMBOSS needle script, which is called internally within the DockQ script. In cases where this alignment failed, often due to insufficient sequence similarity between the native and model chains (especially in cases where short peptides were experimentally solved), the DockQ script did not produce a score, and these pairs were discarded. The highest DockQ score obtained from the three predictions was recorded as the final DockQ score, measuring the interface similarity between the ground truth PDB structure and the AF-M model.

#### Fetching protein sequences

Unless otherwise stated, all protein sequences used for a particular protein represent the full-length isoform sequence reported by the UniProt database at the time of sequence retrieval. To make sequences compatible with AF-M, any non-canonical amino acids not among the standard 20 were removed.

#### Homology reduction of training and testing datasets

Once all contact positive (C+) protein pairs were identified across all 5 datasets used for training and testing we then identified homologous pairs and then randomly selected one pair from a homology set and removed all others. Homologous pairs were identified by first clustering the reviewed human proteome using MMSeqs2^79^ and requiring 50% overlap and 30% sequence similarity. After protein clustering, every protein in every pair was assigned to a cluster id. Homologues pairs were then identified by finding pairs where both proteins in the pair were in the same clusters as the two proteins in another pair.

#### Identifying Homologous pairs in the PDB RefSet and PDB pairs in the AF-M Training set

PDB protein entries were first retrieved by downloading https://files.wwpdb.org/pub/pdb/derived_data/pdb_seqres.txt.gz and then filtering out non-protein entries by entity type. We then used MMSeqs2 to create a map between every reviewed human protein and homologous PDB chains at 30% sequence similarity using the following command: mmseqs easy-search hs_proteome_uniprot_id_map.fasta pdb_protein_entries.fasta 2024119_hs_pdb_vs_uniprot_db_v3.txt tmp -s 7.5 --min-seq-id 0.3 --min-aln-len 6 -e 0.1 --format-mode 4 --exhaustive-search

This MMSeq2 mapping output was then read in via Python and for every pair in our RefSet^PDB^ we identified those PDB entries were both proteins contained interacting homologs. Deposit dates for those entries were then fetched from the PDB API and any pair with a homologous pair deposited before the 2021-09-30 AF-M v2.3 training cutoff was considered to have a homolog in the AF-M 2.3 training set. In a handful of instances (n = 6) this procedure failed to identify any structures containing the pair which is false given that we sourced pairs from the PDB. Investigating these cases revealed that failure occurred because one or more partners was only present as a short peptide in the PDB structure which in turn caused MMSeqs2 to discard mappings to a UniProt entry due to our maximum e-value cutoff. For the purposes of our homolog analysis of SPOC performance we excluded these failed PDB pairs.

#### Random protein sampling

We randomly sampled proteins by randomly shuffling a list of UniProt IDs from the reviewed canonical human proteins downloaded from UniProt and taking the top N ids from this list.

#### Extracting and consolidating cross links from publications

We found 20 publications that performed large-scale cross-linking mass spectrometry (XLMS) studies on the scale of whole proteomes or organelles and provided an easily accessible table of identified crosslinks (Table S2). In cases where cross-links were in mouse proteins, we used the UniProt ID mapping tool^80^ to map mouse UniProt IDs to gene symbols to canonical human UniProt IDs. The mouse and human sequences were aligned using the BioPython Align package, and the crosslinked residue numbers were mapped from mouse coordinates to human coordinates. We discarded any crosslinks where this mapping process failed on the ID or residue level. After collecting all unique residue cross-linking pairs, they were deduplicated first on the level of residues and then on the level of protein pairs. We additionally performed random trimming of over-represented proteins and protein classes. This was done by iteratively identifying the most-common protein across all pairs, randomly selecting all but 28 pairs containing that protein, and then removing them from the list. This process was repeated until no protein was represented more than 28 times across all pairs. After this process, all histone proteins were identified using a list of histone identifiers and randomly removed until histone containing pairs represented only 1% of the final XLMS dataset.

#### Random forest training and testing

We used the RandomForestClassifier package from the scikit-learn to train our random forest (RF) models. We implemented a procedure that combined a grid search of random forest hypermeters along with iterative pruning of features with low GINI importance scores to generate classifiers using a minimal number of features. Our grid search was based on the following parameters: param_grid = {‘n_estimators’: [150, 200, 250],’max_depth’: [3, 5, 7],’min_samples_split’: [2, 5],’bootstrap’: [True, False],’criterion’: [‘gini’,’log_loss’]}. Hyperparameter optimization was performed over K-fold test, train splitting with n = 3 across the training set (Table S6) with the goal of selecting the combination of settings that produced a RF achieving the highest AUC. After each round of hyper-parameter tuning, features were ranked from most to least important via their GINI scores and all features with GINI scores < 0.01 were discarded. The process was then repeated with the reduced feature set until feature pruning ended because no more features with GINI scores below 0.01 were identified. For the structural classifier, we started with a set of 42 features that were reduced to 24 via pruning (Table S3, tabs StructuralC_pre_pruning and StructuralC_post_pruning, respectively). Similarly, SPOC was initially supplied with 56 features and ended up with a final set of 20 features after pruning (Table S3, tabs SPOC_pre_pruning and SPOC_post_pruning ). Otherwise, we used the default values specified by scikit-learn. Data was randomly split, with 75% of pairs from each reference set selected for training while the remaining 25% were used for testing. ROC curve visualizations and AUCs were generated via the roc_curve, AUC functions imported from the sklearn.metrics package. To generate FDR recall plots, we randomly sub-sampled from the held back test data (n = 10 independent times) and constructed test sets with specific ratios of negative to positive examples ranging from ratios of 1:1 to 1:128. In the 1:1 case this corresponded to 714 P to 714 N test pairs while in the 1:128 case, 24 P and 2,912 (all) N pairs were used.

#### AlphaMissense data processing

Data was downloaded from the human proteome-wide precomputed amino acid substitution data table hosted at (https://console.cloud.google.com/storage/browser/dm_alphamissense;tab=objects?pli=1&prefix=&forceOnObjectsSortingFiltering=false). For each residue position, we averaged the AlphaMissense score across all 19 possible missense variants predicted to produce a single, per-residue missense score. These values were then loaded into a JSON dictionary where each key is a UniProt ID that points to a numeric vector (with a length equivalent to the amino acid count of the protein) where each entry is a number from 0 to 100 that represents the averaged missense score predicted for mutating the residue at the corresponding position to a different amino acid.

#### RNA Coexpression data

mRNA co-expression data for human proteins was downloaded from the online web repository coexpressDB (https://zenodo.org/record/6861444/files/Hsa-u.v22-05.G16651-S245698.combat_pca.subagging.z.d.zip). This download returns a folder with files for each gene such that the name of the file is the ENTREZ gene ID. For every protein/gene we sorted by co-expression score (high to low) and took the top 500 pairs before mapping. ENTREZ gene IDs were then mapped to canonical UniProt entry names using the UniProt mapping tool. Pairs where the mapping process failed were discarded. Score values were used as supplied by the database.

#### DEPMAP data

CRISPR KO gene effect data was downloaded from the online resource DEPMAP (https://depmap.org/portal/download/custom/). Every protein was converted into a DEPMAP vector of length n= 1,095 (n = # profiled cell lines) where every entry/dimension in the vector corresponds to the Chronos output (gene effect) for that gene in a cell line.

#### BioGRID ORCS data processing

CRISPR KO data for studies conducted in human cell lines was downloaded as a series of files from the BioGRID file repository (https://downloads.thebiogrid.org/File/BioGRID-ORCS/Release-Archive/BIOGRID-ORCS-1.1.15/BIOGRID-ORCS-ALL-homo_sapiens-1.1.15.screens.tar.gz). Every gene was mapped to a canonical UniProt ID and for each gene its appearance across all the CRISPR screens was converted into binary vectors of length n=1,243 (n = # of screens) where each index represents whether that gene was considered a “hit” (0 = no hit, 1 = hit) by the criterion employed by a specific screen.

#### BioGRID data

BioGRID release 4.4.225 interaction data was downloaded as a tab delimited file on August 29, 2023 using the link supplied by the online repository at: https://downloads.thebiogrid.org/File/BioGRID/Release-Archive/BIOGRID-4.4.225/BIOGRID-ALL-4.4.225.mitab.zip. Interactions were then filtered for human only (taxid:9606). Human protein pairs were then identified using UniProt IDs included in the file. The number of times a unique human pair was found in this file was then used as the biogrid_detect_count feature and encoded into a nested dictionary JSON file where uniprot ids are used as keys and point to the detect count value.

#### DeepLoc2 protein localization predictions

To have uniform localization information for proteins that went beyond standard and incomplete annotations, we utilized predictions from the DeepLoc 2.0 protein sequence transformer model. We downloaded (https://services.healthtech.dtu.dk/cgi-bin/sw_request?software=deeploc&version=2.0&packageversion=2.0&platform=All) and installed a local copy of DeepLoc 2.0. After installation, we inputted a FASTA file containing all canonical SwissProt reviewed sequences for the human proteome downloaded from UniProt. We then used the ESDM1B “fast” model to predict individual localization probabilities split across 10 different possible categories for all sequences. These values were then loaded and stored in a JSON dictionary where each key is a UniProt ID that points to a numeric vector with the 10 localization probabilities output by DeepLoc 2.0.

#### H5 protein embeddings

Per-protein embeddings (vectors of length 1024) were retrieved for all reviewed UniProtKB Swiss-Prot human entries via download from UniProt (https://ftp.UniProt.org/pub/databases/UniProt/current_release/knowledgebase/embeddings/UP000005640_9606/per-protein.h5).

#### STRINGDB scores

All human protein association scores were downloaded from the STRINGv12 database at (https://stringdb-downloads.org/download/protein.links.detailed.v12.0/9606.protein.links.detailed.v12.0.txt.gz). Each entry in the file lists a pair of proteins identified by their STRINGDB ID consisting of the taxon ID (9606 for humans) concatenated with an ENSEMBL protein id. These ENSEMBL protein ids were mapped to UniProt IDs using UniProt’s mapping API. In cases where this mapping yielded non-canonical UniProt IDs or non-SwissProt entries, these ENSEMBL protein ids were mapped to genes and then each gene was mapped to the canonical SwissProt UniProt ID.

#### Replisome structural model

An AF-M prediction of the pol ε holoenzyme (POLE1-POLE2-POLE3-POLE4; only POLE1 shown) was aligned on the C-terminal, non-catalytic lobe of POLE1 in a cryo-EM replisome structure (PDB: 7PLO) using ChimeraX^88^. To model the primer template, the structure of yeast POLE1 catalytic domain with a primer template (PDB: 4M8O) was aligned to the catalytic domain of the AF-M POLE1 structure prediction from the pol ε holoenzyme above. The catalytic domain of POLE1(residues 1–1180), CTF18, CTF8, DCC1 were folded, in which POLE1 made extensive contacts with the CTF18-CTF8-DCC1 complex. The resulting structure was also aligned on POLE1 of the pol ε holoenzyme. Separately, MCM3, MCM7, and CTF18 (only well-ordered residues 281–865) were folded together. MCM3 and the N-terminal lobe of MCM7 (residues 1–319) were deleted, and the remaining C-terminal lobe of MCM7 and CTF18 were aligned on MCM7 from 7PLO.

### QUANTIFICATION AND STATISTICAL ANALYSIS

All graphs display the number of data points presented and analyzed. Ranking data was analyzed using Wilcoxon signed-rank tests as implemented in the scipy.stats.wilcoxon Python function.

### ADDITIONAL RESOURCES

The genome maintenance dataset is available for interactive browsing online at predictomes.org.

## Supplementary Material

1 Table S1: Table of ranking experiment analyses, Related to Figure 1

2 Table S2: Table of XLMS data sources, Related to Figure 2

3 Table S3: Table of classifier features explored, Related to STAR methods

4 Table S4: Table of analysis of the GM predictomes dataset, Related to Figure 5

5 Table S5: Table of metrics for predictions of replication hypotheses, Related to Figure 7

6 Table S6: Table of data for all train/test set pairs, Related to Figure 2 and STAR Methods

7

## Figures and Tables

**KEY RESOURCES TABLE:** 

REAGENT or RESOURCE	SOURCE	IDENTIFIER
**Deposited data**		
Analysis results	This paper	10.5281/zenodo.14641589
All AlphaFold multimer predictions	This paper	10.15785/SBGRID/1155
Whole human proteome AlphaMissense values	Cheng et al.^81^	https://zenodo.org/records/8208688
StringDB values for human protein pairs	Von Mering et al.^57^	https://string-db.org/cgi/download
BioGRID and BioORCS data for human proteins	Oughtred et al.^84^	https://downloads.thebiogrid.org/BioGRID
ProtT5 protein embeddings for all reviewed human proteins	UniProt	https://www.uniprot.org/help/downloads#embeddings
CoExpress DB mRNA co-expression values for all human genes	Obayashi et al.^85^	https://zenodo.org/records/6861444
DepMap data	Tsherniak et al.^87^	https://depmap.org/portal/data_page/
All protein sequences in the PDB	Berman et al.^14^	https://www.rcsb.org/downloads/fasta
**Software and algorithms**		
ColabFold	Mirdita et al.^77^	https://github.com/sokrypton/ColabFold
AlphaFold Multimer	Evans et al.^12^	https://github.com/google-deepmind/alphafold
Visual Studio Code	Microsoft	https://code.visualstudio.com/
GoogleColab	Google	https://colab.research.google.com/
Sci-kit learn version 1.2.2	Scikit learn maintained team	https://scikit-learn.org/
BioPython version 1.83	Cock et al.^82^	https://biopython.org/
DockQ	Basu et al.^83^	https://github.com/bjomwallner/DockQ
pDockQ	Bryant et al.^34^	https://gitlab.com/ElofssonLab/FoldDock
pDockQv2	Zhu et al.^36^	https://gitlab.com/ElofssonLab/afm-benchmark
DeepLoc 2.0	Thumuluri et al.^86^	https://services.healthtech.dtu.dk/services/DeepLoc-2.0/
MMseqs2	Steinegger et al.^79^	https://github.com/soedinglab/MMseqs2
UniProt ID Mapping tool	Zaru et al.^80^	https://www.uniprot.org/id-mapping
ChimeraX	Meng et al.^88^	https://www.cgl.ucsf.edu/chimerax/
MolStar	Sehnal et al.^89^	https://github.com/molstar/molstar
RCSB Protein Data Bank API	Rose et al.^90^	https://data.rcsb.org
ChatGPT	OpenAI	https://chat.openai.com/
Analysis code and results	This paper	10.5281/zenodo.14641589
SPOC command line tool	This paper	https://github.com/walterlab-HMS/SPOC
SPOC command line tool v1 GitHub archive	This paper	10.5281/zenodo.14768322
**Other**		
Online webportal for viewing AlphaFold multimer screen datasets	This paper	https://predictomes.org
A100 40GB GPU rental	Lambda Labs	https://lambdalabs.com/

## Highlights

SPOC classifier identifies functional AlphaFold-Multimer structure predictions

Proteome-wide in silico screening with AlphaFold-Multimer enabled by SPOC classifier

Curated database of 40,000 binary structure predictions in genome maintenance

User-friendly website predictomes.org to browse predictions and build hypotheses

## References

[1] Alberts B (1998). The Cell as a Collection of Protein Machines: Preparing the Next Generation of Molecular Biologists. Cell 92, 291–294. 10.1016/S0092-8674(00)80922-8. 9476889

[2] Luck K, Kim DK, Lambourne L, Spirohn K, Begg BE, Bian W, Brignall R, Cafarelli T, Campos-Laborie FJ, Charloteaux B, (2020). A reference map of the human binary protein interactome. Nature 2020 580:7803 580, 402–408. 10.1038/s41586-020-2188-x. PMC7169983 32296183

[3] Walport LJ, Low JKK, Matthews JM, and MacKay JP (2021). The characterization of protein interactions – what, how and how much? Chem Soc Rev 50, 12292–12307. 10.1039/D1CS00548K. 34581717

[4] Choi SG, Richardson A, Lambourne L, Hill DE, and Vidal M (2018). Protein Interactomics by Two-Hybrid Methods. Methods in Molecular Biology 1794, 1–14. 10.1007/978-1-4939-7871-7_1. 29855947 PMC6948107

[5] Huttlin EL, Bruckner RJ, Navarrete-Perea J, Cannon JR, Baltier K, Gebreab F, Gygi MP, Thornock A, Zarraga G, Tam S, (2021). Dual proteome-scale networks reveal cell-specific remodeling of the human interactome. Cell 184, 3022–3040.e28. 10.1016/J.CELL.2021.04.011/ATTACHMENT/9D45895F-3A8F-4F89-9279-12FF7DCE96A4/MMC7.ZIP. 33961781 PMC8165030

[6] Wan C, Borgeson B, Phanse S, Tu F, Drew K, Clark G, Xiong X, Kagan O, Kwan J, Bezginov A, (2015). Panorama of ancient metazoan macromolecular complexes. Nature 2015 525:7569 525, 339–344. 10.1038/nature14877. PMC5036527 26344197

[7] Sinn LR, Giese SH, Stuiver M, and Rappsilber J (2022). Leveraging Parameter Dependencies in High-Field Asymmetric Waveform Ion-Mobility Spectrometry and Size Exclusion Chromatography for Proteome-wide Cross-Linking Mass Spectrometry. Anal Chem 94, 4627–4634. 10.1021/ACS.ANALCHEM.1C04373/ASSET/IMAGES/LARGE/AC1C04373_0004.JPEG. 35276035 PMC8943524

[8] Bordoli L, Kiefer F, Arnold K, Benkert P, Battey J, and Schwede T (2008). Protein structure homology modeling using SWISS-MODEL workspace. Nature Protocols 2009 4:1 4, 1–13. 10.1038/nprot.2008.197. 19131951

[9] Zhang QC, Petrey D, Deng L, Qiang L, Shi Y, Thu CA, Bisikirska B, Lefebvre C, Accili D, Hunter T, (2012). Structure-based prediction of protein–protein interactions on a genome-wide scale. Nature 2012 490:7421 490, 556–560. 10.1038/nature11503. PMC3482288 23023127

[10] Pierce BG, Hourai Y, and Weng Z (2011). Accelerating Protein Docking in ZDOCK Using an Advanced 3D Convolution Library. PLoS One 6, e24657. 10.1371/JOURNAL.PONE.0024657. 21949741 PMC3176283

[11] Meyer MJ, Beltrán JF, Liang S, Fragoza R, Rumack A, Liang J, Wei X, and Yu H (2018). Interactome INSIDER: a structural interactome browser for genomic studies. Nature Methods 2018 15:2 15, 107–114. 10.1038/nmeth.4540. PMC6026581 29355848

[12] Evans R, O’Neill M, Pritzel A, Antropova N, Senior A, Green T, Žídek A, Bates R, Blackwell S, Yim J, (2022). Protein complex prediction with AlphaFold-Multimer. bioRxiv, 2021.10.04.463034. 10.1101/2021.10.04.463034.

[13] Baek M, DiMaio F, Anishchenko I, Dauparas J, Ovchinnikov S, Lee GR, Wang J, Cong Q, Kinch LN, Dustin Schaeffer R, (2021). Accurate prediction of protein structures and interactions using a three-track neural network. Science (1979) 373, 871–876. 10.1126/SCIENCE.ABJ8754/SUPPL_FILE/ABJ8754_MDAR_REPRODUCIBILITY_CHECKLIST.PDF. PMC7612213 34282049

[14] Berman HM, Westbrook J, Feng Z, Gilliland G, Bhat TN, Weissig H, Shindyalov IN, and Bourne PE (2000). The Protein Data Bank. Nucleic Acids Res 28, 235–242. 10.1093/NAR/28.1.235. 10592235 PMC102472

[15] Wang B, Vartak R, Zaltsman Y, Zar Z, Naing C, Hennick KM, Polacco BJ, Bashir A, Eckhardt M, Bouhaddou M, (2024). A foundational atlas of autism protein interactions reveals molecular convergence. bioRxiv, 2023.12.03.569805. 10.1101/2023.12.03.569805.

[16] Banhos Danneskiold-Sams E N, Kavi D, Jude KM, Nissen SB, Wat LW, Coassolo L, Zhao M, Asae Santana-Oikawa G, Broido BB, Garcia KC, (2023). Rapid and accurate deorphanization of ligand-receptor pairs using AlphaFold. Preprint, 10.1101/2023.03.16.531341 . PMC12147870 39541981

[17] Burke DF, Bryant P, Barrio-Hernandez I, Memon D, Pozzati G, Shenoy A, Zhu W, Dunham AS, Albanese P, Keller A, (2023). Towards a structurally resolved human protein interaction network. Nature Structural & Molecular Biology 2023 30:2 30, 216–225. 10.1038/s41594-022-00910-8. PMC9935395 36690744

[18] Olsvik HL, and Johansen T (2023). AlphaFold-multimer predicts ATG8 protein binding motifs crucial for autophagy research. PLoS Biol 21, e3002002. 10.1371/JOURNAL.PBIO.3002002. 36848650 PMC9907820

[19] Sifri C, Hoeg L, Durocher D, and Setiaputra D (2023). An AlphaFold2 map of the 53BP1 pathway identifies a direct SHLD3–RIF1 interaction critical for shieldin activity. EMBO Rep 24. 10.15252/EMBR.202356834/SUPPL_FILE/EMBR202356834-SUP-0006-SDATAEV.ZIP. PMC10398656 37306046

[20] O’Reilly FJ, Graziadei A, Forbrig C, Bremenkamp R, Charles K, Lenz S, Elfmann C, Fischer L, Stülke J, and Rappsilber J (2023). Protein complexes in cells by AI -assisted structural proteomics. Mol Syst Biol 19, 11544. 10.15252/MSB.202311544/SUPPL_FILE/MSB202311544-SUP-0009-DATASETEV8.XLSX. PMC10090944 36815589

[21] Yu D, Chojnowski G, Rosenthal M, and Kosinski J (2023). AlphaPulldown—a python package for protein–protein interaction screens using AlphaFold-Multimer. Bioinformatics 39. 10.1093/BIOINFORMATICS/BTAC749. PMC9805587 36413069

[22] Schweke H, Pacesa M, Levin T, Goverde CA, Kumar P, Duhoo Y, Dornfeld LJ, Dubreuil B, Georgeon S, Ovchinnikov S, (2024). An atlas of protein homo-oligomerization across domains of life. Cell 187, 999–1010.e15. 10.1016/J.CELL.2024.01.022. 38325366

[23] Lim Y, Tamayo-Orrego L, Schmid E, Tarnauskaite Z, Kochenova OV, Gruar R, Muramatsu S, Lynch L, Schlie AV, Carroll PL, (2023). In silico protein interaction screening uncovers DONSON’s role in replication initiation. Science (1979) 381, eadi3448. 10.1126/science.adi3448. PMC10801813 37590370

[24] Deneke VE, Blaha A, Lu Y, Suwita JP, Draper JM, Phan CS, Panser K, Schleiffer A, Jacob L, Humer T, (2024). A conserved fertilization complex bridges sperm and egg in vertebrates. Cell. 10.1016/j.cell.2024.09.035. 39423812

[25] Jeppesen M, and André I (2023). Accurate prediction of protein assembly structure by combining AlphaFold and symmetrical docking. Nature Communications 2023 14:1 14, 1–13. 10.1038/s41467-023-43681-6. PMC10719378 38092742

[26] Bryant P, Pozzati G, Zhu W, Shenoy A, Kundrotas P, and Elofsson A (2022). Predicting the structure of large protein complexes using AlphaFold and Monte Carlo tree search. Nature Communications 2022 13:1 13, 1–14. 10.1038/s41467-022-33729-4. PMC9556563 36224222

[27] Yin R, Feng BY, Varshney A, and Pierce BG (2022). Benchmarking AlphaFold for protein complex modeling reveals accuracy determinants. Protein Sci 31. 10.1002/PRO.4379. PMC9278006 35900023

[28] Zhu W, Shenoy A, Kundrotas P, and Elofsson A (2023). Evaluation of AlphaFold-Multimer prediction on multi-chain protein complexes. Bioinformatics 39. 10.1093/BIOINFORMATICS/BTAD424. PMC10348836 37405868

[29] Lim Y, Tamayo-Orrego L, Schmid E, Tarnauskaite Z, Kochenova OV, Gruar R, Muramatsu S, Lynch L, Schlie AV, Carroll PL, (2023). In silico protein interaction screening uncovers DONSON’s role in replication initiation. Science (1979) 381, eadi3448. 10.1126/science.adi3448. PMC10801813 37590370

[30] Hashimoto Y, Sadano K, Miyata N, Ito H, and Tanaka H (2023). Novel role of DONSON in CMG helicase assembly during vertebrate DNA replication initiation. EMBO J 42. 10.15252/EMBJ.2023114131. PMC10476173 37458194

[31] Kingsley G, Skagia A, Passaretti P, Fernandez-Cuesta C, Reynolds-Winczura A, Koscielniak K, and Gambus A (2023). DONSON facilitates Cdc45 and GINS chromatin association and is essential for DNA replication initiation. Nucleic Acids Res 51, 9748–9763. 10.1093/NAR/GKAD694. 37638758 PMC10570026

[32] Cvetkovic MA, Passaretti P, Butryn A, Reynolds-Winczura A, Kingsley G, Skagia A, Fernandez-Cuesta C, Poovathumkadavil D, George R, Chauhan AS, (2023). The structural mechanism of dimeric DONSON in replicative helicase activation. Mol Cell 83, 4017–4031.e9. 10.1016/J.MOLCEL.2023.09.029. 37820732 PMC7616792

[33] Lim Y, Tamayo-Orrego L, Schmid E, Tarnauskaite Z, Kochenova OV, Gruar R, Muramatsu S, Lynch L, Schlie AV, Carroll PL, (2023). In silico protein interaction screening uncovers DONSON’s role in replication initiation. Science (1979) 381, eadi3448. 10.1126/science.adi3448. PMC10801813 37590370

[34] Bryant P, Pozzati G, and Elofsson A (2022). Improved prediction of protein-protein interactions using AlphaFold2. Nature Communications 2022 13:1 13, 1–11. 10.1038/s41467-022-28865-w. PMC8913741 35273146

[35] Kokic G, Yakoub G, van den Heuvel D, Wondergem AP, van der Meer PJ, van der Weegen Y, Chernev A, Fianu I, Fokkens TJ, Lorenz S, (2024). Structural basis for RNA polymerase II ubiquitylation and inactivation in transcription-coupled repair. Nature Structural & Molecular Biology 2024 31:3 31, 536–547. 10.1038/s41594-023-01207-0. PMC10948364 38316879

[36] Zhu W, Shenoy A, Kundrotas P, and Elofsson A (2023). Evaluation of AlphaFold-Multimer prediction on multi-chain protein complexes. Bioinformatics 39. 10.1093/BIOINFORMATICS/BTAD424. PMC10348836 37405868

[37] Frénay B, and Verleysen M (2014). Classification in the presence of label noise: A survey. IEEE Trans Neural Netw Learn Syst 25, 845–869. 10.1109/TNNLS.2013.2292894. 24808033

[38] Luck K, Kim DK, Lambourne L, Spirohn K, Begg BE, Bian W, Brignall R, Cafarelli T, Campos-Laborie FJ, Charloteaux B, (2020). A reference map of the human binary protein interactome. Nature 2020 580:7803 580, 402–408. 10.1038/s41586-020-2188-x. PMC7169983 32296183

[39] Venkatesan K, Rual JF, Vazquez A, Stelzl U, Lemmens I, Hirozane-Kishikawa T, Hao T, Zenkner M, Xin X, Goh K Il, (2008). An empirical framework for binary interactome mapping. Nature Methods 2008 6:1 6, 83–90. 10.1038/nmeth.1280. PMC2872561 19060904

[40] Stumpf MPH, Thorne T, De Silva E, Stewart R, Hyeong JA, Lappe M, and Wiuf C (2008). Estimating the size of the human interactome. Proc Natl Acad Sci U S A 105, 6959–6964. 10.1073/PNAS.0708078105/SUPPL_FILE/0708078105SI.PDF. 18474861 PMC2383957

[41] Tompa P, Davey NE, Gibson TJ, and Babu MM (2014). A Million Peptide Motifs for the Molecular Biologist. Mol Cell 55, 161–169. 10.1016/J.MOLCEL.2014.05.032. 25038412

[42] O’Reilly FJ, and Rappsilber J (2018). Cross-linking mass spectrometry: methods and applications in structural, molecular and systems biology. Nature Structural & Molecular Biology 2018 25:11 25, 1000–1008. 10.1038/s41594-018-0147-0. 30374081

[43] Basu S, and Wallner B (2016). DockQ: A Quality Measure for Protein-Protein Docking Models. PLoS One 11, e0161879. 10.1371/JOURNAL.PONE.0161879. 27560519 PMC4999177

[44] Breiman L (2001). Random Forests. Mach Learn 12343 LNCS, 5–32. 10.1007/978-3-030-62008-0_35.

[45] Dunham B, and Ganapathiraju MK (2021). Benchmark Evaluation of Protein–Protein Interaction Prediction Algorithms. Molecules 2022, Vol. 27, Page 41 27, 41. 10.3390/MOLECULES27010041. PMC8746451 35011283

[46] Tsherniak A, Vazquez F, Montgomery PG, Weir BA, Kryukov G, Cowley GS, Gill S, Harrington WF, Pantel S, Krill-Burger JM, (2017). Defining a Cancer Dependency Map. Cell 170, 564–576.e16. 10.1016/j.cell.2017.06.010. 28753430 PMC5667678

[47] Obayashi T, Kodate S, Hibara H, Kagaya Y, and Kinoshita K (2023). COXPRESdb v8: an animal gene coexpression database navigating from a global view to detailed investigations. Nucleic Acids Res 51, D80–D87. 10.1093/NAR/GKAC983. 36350658 PMC9825429

[48] Elnaggar A, Heinzinger M, Dallago C, Rihawi G, Wang Y, Jones L, Gibbs T, Feher T, Angerer C, Steinegger M, (2020). ProtTrans: Towards Cracking the Language of Life’s Code Through Self-Supervised Deep Learning and High Performance Computing. bioRxiv 14.

[49] Thumuluri V, Almagro Armenteros JJ, Johansen AR, Nielsen H, and Winther O (2022). DeepLoc 2.0: multi-label subcellular localization prediction using protein language models. Nucleic Acids Res 50, W228–W234. 10.1093/NAR/GKAC278. 35489069 PMC9252801

[50] Oughtred R, Rust J, Chang C, Breitkreutz BJ, Stark C, Willems A, Boucher L, Leung G, Kolas N, Zhang F, (2021). The BioGRID database: A comprehensive biomedical resource of curated protein, genetic, and chemical interactions. Protein Science 30, 187–200. 10.1002/PRO.3978. 33070389 PMC7737760

[51] Räschle M, Smeenk G, Hansen RK, Temu T, Oka Y, Hein MY, Nagaraj N, Long DT, Walter JC, Hofmann K, (2015). Proteomics reveals dynamic assembly of Repair complexes during bypass of DNA cross-links. Science (1979) 348. 10.1126/science.1253671. PMC5331883 25931565

[52] Huang W, Qiu F, Zheng L, Shi M, Shen M, Zhao X, and Xiang S (2023). Structural insights into Rad18 targeting by the SLF1 BRCT domains. Journal of Biological Chemistry 299, 105288. 10.1016/j.jbc.2023.105288. 37748650 PMC10598736

[53] Cho T, Hoeg L, Setiaputra D, and Durocher D (2024). NFATC2IP is a mediator of SUMO-dependent genome integrity. Genes Dev. 10.1101/gad.350914.123. PMC11065178 38503515

[54] Mevissen TET, Kümmecke M, Schmid EW, Farnung L, and Walter JC (2024). STK19 positions TFIIH for cell-free transcription-coupled DNA repair. Cell 0. 10.1016/J.CELL.2024.10.020. PMC11645862 39547228

[55] Mevissen TET, Kümmecke M, Schmid EW, Farnung L, and Walter JC (2024). STK19 positions TFIIH for cell-free transcription-coupled DNA repair. bioRxiv, 2024.07.22.604623. 10.1101/2024.07.22.604623. PMC11645862 39547228

[56] Kochenova OV, D’Alessandro G, Pilger D, Schmid E, Richards SL, Garcia MR, Jhujh SS, Voigt A, Gupta V, Carnie CJ, (2024). USP37 prevents premature disassembly of stressed replisomes by TRAIP. bioRxiv, 2024.09.03.611025. 10.1101/2024.09.03.611025. PMC12177040 40533495

[57] von Mering C, Jensen LJ, Snel B, Hooper SD, Krupp M, Foglierini M, Jouffre N, Huynen MA, and Bork P (2005). STRING: known and predicted protein–protein associations, integrated and transferred across organisms. Nucleic Acids Res 33, D433–D437. 10.1093/NAR/GKI005. 15608232 PMC539959

[58] Piwko W, Mlejnkova LJ, Mutreja K, Ranjha L, Stafa D, Smirnov A, Brodersen MM, Zellweger R, Sturzenegger A, Janscak P, (2016). The MMS22L–TONSL heterodimer directly promotes RAD51-dependent recombination upon replication stress. EMBO J 35, 2584–2601. 10.15252/EMBJ.201593132/SUPPL_FILE/EMBJ201593132-SUP-0001-EVFIGS.PDF. 27797818 PMC5283591

[59] del Toro N, Shrivastava A, Ragueneau E, Meldal B, Combe C, Barrera E, Perfetto L, How K, Ratan P, Shirodkar G, (2022). The IntAct database: efficient access to fine-grained molecular interaction data. Nucleic Acids Res 50, D648–D653. 10.1093/NAR/GKAB1006. 34761267 PMC8728211

[60] Adam S, Rossi SE, Moatti N, De Marco Zompit M, Xue Y, Ng TF, Álvarez-Quilón A, Desjardins J, Bhaskaran V, Martino G, (2021). The CIP2A–TOPBP1 axis safeguards chromosome stability and is a synthetic lethal target for BRCA-mutated cancer. Nature Cancer 2021 2:12 2, 1357–1371. 10.1038/s43018-021-00266-w. 35121901

[61] Wang J, Okkeri J, Pavic K, Wang Z, Kauko O, Halonen T, Sarek G, Ojala PM, Rao Z, Xu W, (2017). Oncoprotein CIP 2A is stabilized via interaction with tumor suppressor PP 2A/B56. EMBO Rep 18, 437–450. 10.15252/EMBR.201642788/SUPPL_FILE/EMBR201642788-SUP-0003-SDATAEV.ZIP. 28174209 PMC5331240

[62] Fernandes JB, Duhamel M, Seguéla-Arnaud M, Froger N, Girard C, Choinard S, Solier V, De Winne N, De Jaeger G, Gevaert K, (2018). FIGL1 and its novel partner FLIP form a conserved complex that regulates homologous recombination. PLoS Genet 14, e1007317. 10.1371/JOURNAL.PGEN.1007317. 29608566 PMC5897033

[63] Tsaridou S, and van Vugt MATM (2024). FIRRM and FIGNL1: partners in the regulation of homologous recombination. Trends in Genetics. 10.1016/J.TIG.2024.02.007. 38494375

[64] O’Donnell L, Panier S, Wildenhain J, Tkach JM, Al-Hakim A, Landry MC, Escribano-Diaz C, Szilard RK, Young JTF, Munro M, (2010). The MMS22L-TONSL Complex Mediates Recovery from Replication Stress and Homologous Recombination. Mol Cell 40, 619–631. 10.1016/J.MOLCEL.2010.10.024. 21055983 PMC3031522

[65] O’Connell BC, Adamson B, Lydeard JR, Sowa ME, Ciccia A, Bredemeyer AL, Schlabach M, Gygi SP, Elledge SJ, and Harper JW (2010). A Genome-wide Camptothecin Sensitivity Screen Identifies a Mammalian MMS22L-NFKBIL2 Complex Required for Genomic Stability. Mol Cell 40, 645–657. 10.1016/J.MOLCEL.2010.10.022. 21055985 PMC3006237

[66] Duro E, Lundin C, Ask K, Sanchez-Pulido L, MacArtney TJ, Toth R, Ponting CP, Groth A, Helleday T, and Rouse J (2010). Identification of the MMS22L-TONSL Complex that Promotes Homologous Recombination. Mol Cell 40, 632–644. 10.1016/J.MOLCEL.2010.10.023. 21055984

[67] Sehnal D, Bittrich S, Deshpande M, Svobodová R, Berka K, Bazgier V, Velankar S, Burley SK, Koča J, and Rose AS (2021). Mol* Viewer: modern web app for 3D visualization and analysis of large biomolecular structures. Nucleic Acids Res 49, W431–W437. 10.1093/NAR/GKAB314. 33956157 PMC8262734

[68] Jones ML, Aria V, Baris Y, and Yeeles JTP (2023). How Pol α-primase is targeted to replisomes to prime eukaryotic DNA replication. Mol Cell 83, 2911–2924.e16. 10.1016/J.MOLCEL.2023.06.035. 37506699 PMC10501992

[69] Huang ME, Le Douarin B, Henry C, and Galibert F (1999). The Saccharomyces cerevisiae protein YJR043C (Pol32) interacts with the catalytic subunit of DNA polymerase α and is required for cell cycle progression in G2/M. Molecular and General Genetics 260, 541–550. 10.1007/S004380050927/METRICS. 9928933

[70] Johansson E, Garg P, and Burgers PMJ (2004). The Pol32 Subunit of DNA Polymerase δ Contains Separable Domains for Processive Replication and Proliferating Cell Nuclear Antigen (PCNA) Binding. Journal of Biological Chemistry 279, 1907–1915. 10.1074/JBC.M310362200. 14594808

[71] Maga G, and Hübscher U (1996). DNA Replication Machinery: Functional Characterization of a Complex Containing DNA Polymerase α, DNA Polymerase δ, and Replication Factor C Suggests an Asymmetric DNA Polymerase Dimer†. Biochemistry 35, 5764–5777. 10.1021/BI952455K. 8639537

[72] Lewis JS, Spenkelink LM, Schauer GD, Yurieva O, Mueller SH, Natarajan V, Kaur G, Maher C, Kay C, O’Donnell ME, (2020). Tunability of DNA Polymerase Stability during Eukaryotic DNA Replication. Mol Cell 77, 17–25.e5. 10.1016/J.MOLCEL.2019.10.005. 31704183 PMC6943181

[73] Stokes K, Winczura A, Song B, De Piccoli G, and Grabarczyk DB (2020). Ctf18-RFC and DNA Pol ϵ form a stable leading strand polymerase/clamp loader complex required for normal and perturbed DNA replication. Nucleic Acids Res 48, 8128–8145. 10.1093/NAR/GKAA541. 32585006 PMC7641331

[74] Rn Wallner B, and Kelso J (2023). AFsample: improving multimer prediction with AlphaFold using massive sampling. Bioinformatics 39. 10.1093/BIOINFORMATICS/BTAD573. PMC10534052 37713472

[75] Lee CY, Hubrich D, Varga JK, Schäfer C, Welzel M, Schumbera E, Djokic M, Strom JM, Schönfeld J, Geist JL, (2024). Systematic discovery of protein interaction interfaces using AlphaFold and experimental validation. Mol Syst Biol 20, 75–97. 10.1038/S44320-023-00005-6/SUPPL_FILE/44320_2023_5_MOESM15_ESM.ZIP. 38225382 PMC10883280

[76] Bret H, Gao J, Zea DJ, Andreani J, and Guerois R (2024). From interaction networks to interfaces, scanning intrinsically disordered regions using AlphaFold2. Nature Communications 2024 15:1 15, 1–14. 10.1038/s41467-023-44288-7. PMC10796318 38238291

[77] Mirdita M, Schütze K, Moriwaki Y, Heo L, Ovchinnikov S, and Steinegger M (2022). ColabFold: making protein folding accessible to all. Nat Methods 19, 679–682. 10.1038/s41592-022-01488-1. 35637307 PMC9184281

[78] Jenkyn-Bedford M, Jones ML, Baris Y, Labib KPM, Cannone G, Yeeles JTP, and Deegan TD (2021). A conserved mechanism for regulating replisome disassembly in eukaryotes. Nature 2021 600:7890 600, 743–747. 10.1038/s41586-021-04145-3. PMC8695382 34700328

[79] Steinegger M, and Söding J (2017). MMseqs2 enables sensitive protein sequence searching for the analysis of massive data sets. Nature Biotechnology 2017 35:11 35, 1026–1028. 10.1038/nbt.3988. 29035372

[80] Zaru R, and Orchard S (2023). UniProt Tools: BLAST, Align, Peptide Search, and ID Mapping. Curr Protoc 3, e697. 10.1002/CPZ1.697. 36943033 PMC10034637

[81] Cheng J, Novati G, Pan J, Bycroft C, Žemgulytė A, Applebaum T, Pritzel A, Wong LH, Zielinski M, Sargeant T, (2023). Accurate proteome-wide missense variant effect prediction with AlphaMissense. Science (1979) 381, eadg7492. 10.1126/science.adg7492. 37733863

[82] Cock PJA, Antao T, Chang JT, Chapman BA, Cox CJ, Dalke A, Friedberg I, Hamelryck T, Kauff F, Wilczynski B, (2009). Biopython: freely available Python tools for computational molecular biology and bioinformatics. Bioinformatics 25, 1422–1423. 10.1093/BIOINFORMATICS/BTP163. 19304878 PMC2682512

[83] Basu S, and Wallner B (2016). DockQ: A Quality Measure for Protein-Protein Docking Models. PLoS One 11, e0161879. 10.1371/JOURNAL.PONE.0161879. 27560519 PMC4999177

[84] Oughtred R, Rust J, Chang C, Breitkreutz BJ, Stark C, Willems A, Boucher L, Leung G, Kolas N, Zhang F, (2021). The BioGRID database: A comprehensive biomedical resource of curated protein, genetic, and chemical interactions. Protein Sci 30, 187–200. 10.1002/PRO.3978. 33070389 PMC7737760

[85] Obayashi T, Kodate S, Hibara H, Kagaya Y, and Kinoshita K (2023). COXPRESdb v8: an animal gene coexpression database navigating from a global view to detailed investigations. Nucleic Acids Res 51, D80–D87. 10.1093/NAR/GKAC983. 36350658 PMC9825429

[86] Thumuluri V, Almagro Armenteros JJ, Johansen AR, Nielsen H, and Winther O (2022). DeepLoc 2.0: multi-label subcellular localization prediction using protein language models. Nucleic Acids Res 50, W228–W234. 10.1093/NAR/GKAC278. 35489069 PMC9252801

[87] Tsherniak A, Vazquez F, Montgomery PG, Weir BA, Kryukov G, Cowley GS, Gill S, Harrington WF, Pantel S, Krill-Burger JM, (2017). Defining a Cancer Dependency Map. Cell 170, 564–576.e16. 10.1016/J.CELL.2017.06.010. 28753430 PMC5667678

[88] Meng EC, Goddard TD, Pettersen EF, Couch GS, Pearson ZJ, Morris JH, and Ferrin TE (2023). UCSF ChimeraX: Tools for structure building and analysis. Protein Science 32, e4792. 10.1002/PRO.4792. 37774136 PMC10588335

[89] Sehnal D, Bittrich S, Deshpande M, Svobodová R, Berka K, Bazgier V, Velankar S, Burley SK, Koča J, and Rose AS (2021). Mol* Viewer: modern web app for 3D visualization and analysis of large biomolecular structures. Nucleic Acids Res 49, W431–W437. 10.1093/NAR/GKAB314. 33956157 PMC8262734

[90] Rose Y, Duarte JM, Lowe R, Segura J, Bi C, Bhikadiya C, Chen L, Rose AS, Bittrich S, Burley SK, (2021). RCSB Protein Data Bank: Architectural Advances Towards Integrated Searching and Efficient Access to Macromolecular Structure Data from the PDB Archive. J Mol Biol 433, 166704. 10.1016/J.JMB.2020.11.003. 33186584 PMC9093041

