# Supplementary material for: Predictomes, a classifier-curated database of AlphaFold-modeled protein-protein interactions
Source: Mol Cell. Author manuscript; Available in PMC 2025 Mar 24. (PMC11931459; doi:10.1016/j.molcel.2025.01.034)
Supplement: 7 [file NIHMS2056204-supplement-7.pdf]

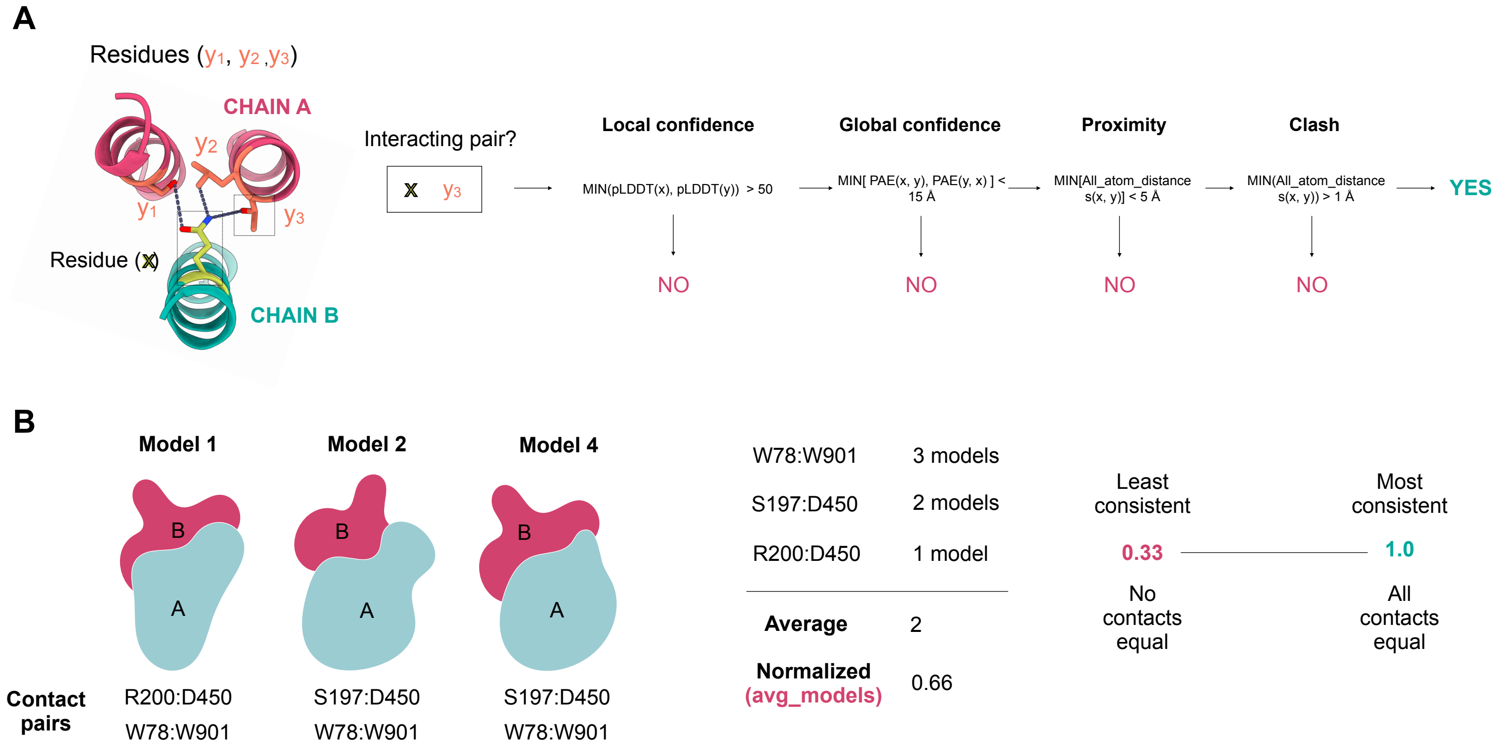

Figure S1. Measuring predicted AF-M interface quality, Related to Figure 1

(A) All our analysis pipelines first identify residues between chains that satisfy the illustrated criteria. Such “C+” pairs are considered valid interfacial contacts and are used for downstream calculations of interface pLDDT, PAE, etc. **(B)** To quantify the agreement among multiple AF-M predictions of a binary complex, we first identify all valid (C+) residue pairs across found in all the predictions and then count how many predictions each unique contact appears in. This count is averaged across all unique, C+ contacts and then normalized by dividing by the number of predicted structures, yielding the “avg_models” score. In the example shown, one contact is common to all three predictions (W78:W901), one is common to two (S197:D450), and one is unique to one pair (R200:D450), yielding avg_models = (3 + 2 + 1)/ 3 * 3 = 0.66. If all three contacts appeared in all three predictions, avg_models would be 1, indicating perfect agreement between all models.

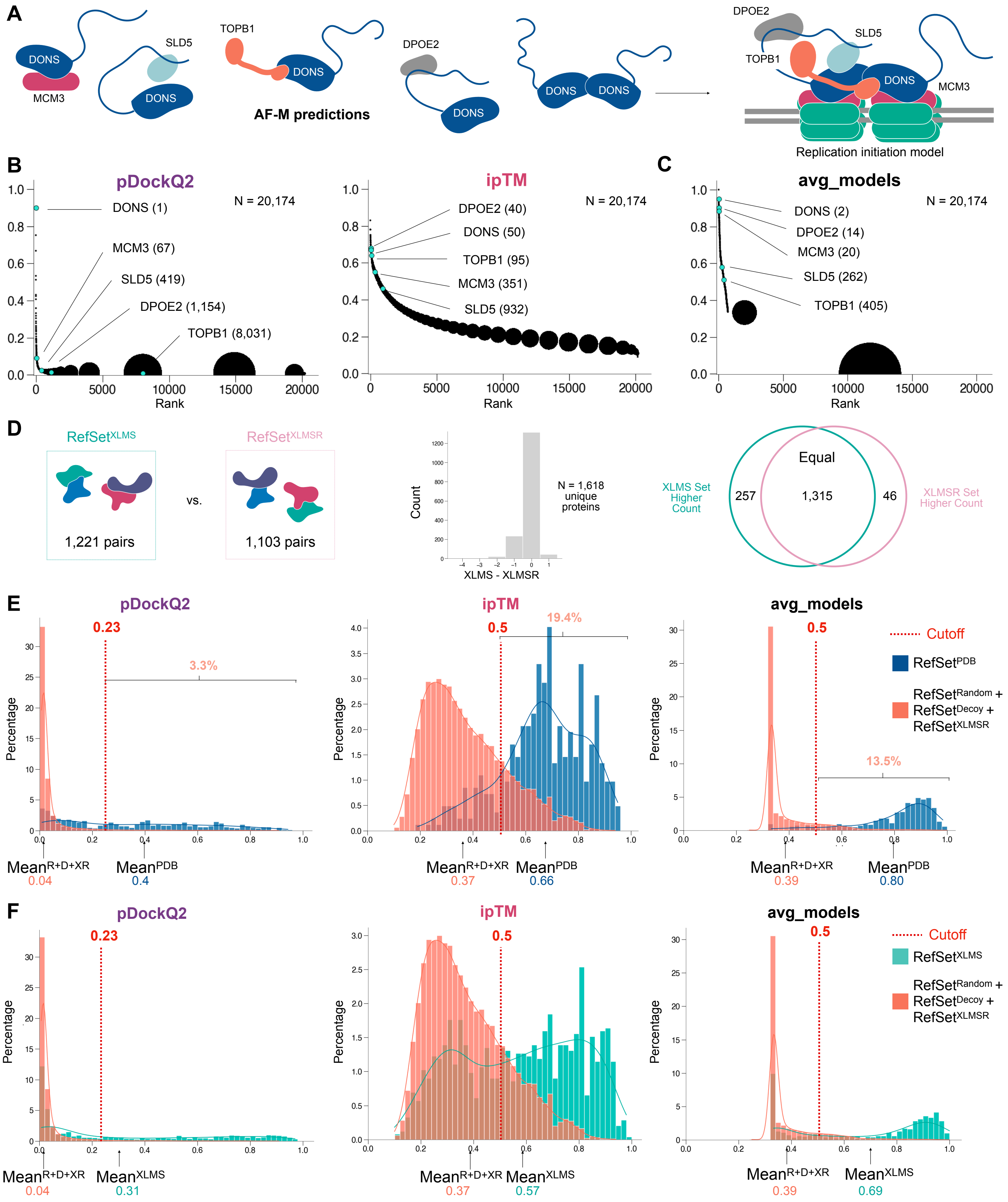

Figure S2: Conventional AF-M confidence metrics have limitations

Figure S2. Conventional AF-M confidence metrics have limitations, Related to Figure 2

(A) A prior screen for DONSON interactors identified 5 proteins (DPOE2, SLD5, TOPB1, and MCM3, and DONS itself) that were shown to be functional DONSON partners. These binary predictions supported a new model of CMG assembly by DONSON. **(B)** We previously used AF-M to screen DONSON against the entire human proteome and ranked the hits using existing confidence metrics. Here, we display the same data (but recalculated using our updated contact criteria; see methods) as rank plots, which show that pDockQ2, and ipTM scatter DONSON's interactors over the top ~1,000-10,000 hits. **(C)** Same as (B) but DONSON hits were ranked by avg_models. **(D)** Scheme illustrating how similar the pairs between the $RefSet^{XLMS}$ and $RefSet^{XLMSR}$ are based on shared proteins between the two sets. **(E)** Distribution of true negative ($RefSet^{Random}$ + $RefSet^{XLMSR}$ + $RefSet^{Decoy}$) and positive ($RefSet^{PDB}$) pairs sorted by pDockQ2, ipTM, and the avg_models scores. Percentage of pairs exceeding the conventional threshold criteria (dotted line) for each metric is shown above the bracket. **(F)** Distribution of true negative ($RefSet^{Random}$ + $RefSet^{XLMSR}$ + $RefSet^{Decoy}$) and training ($RefSet^{XLMS}$) pairs (all C+) sorted by pDockQ2, ipTM, and the avg_models scores.

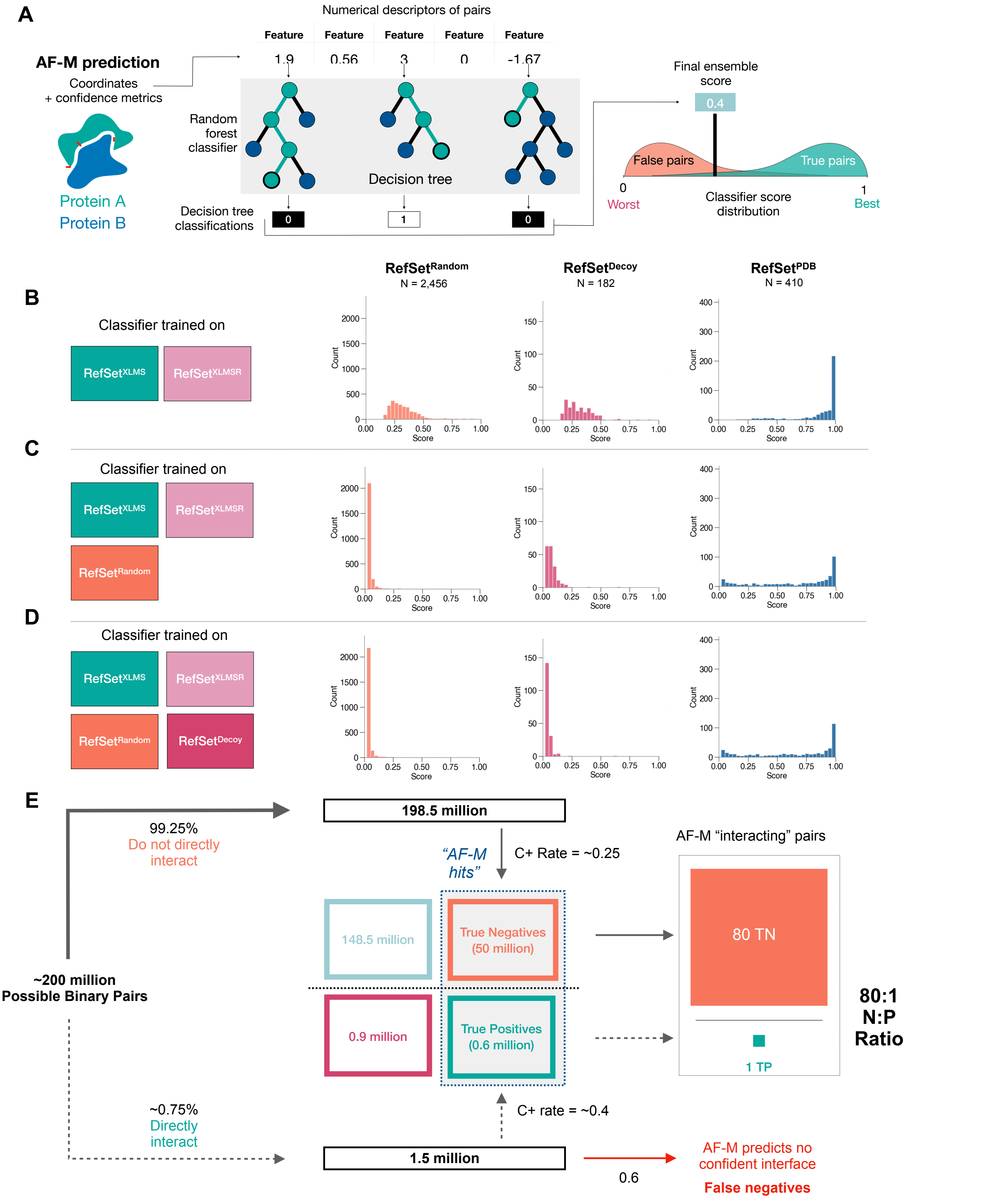

Figure S3. Assessing how dataset composition affects classifier performance characteristics, Related to Figure 3

Figure S3. Assessing how dataset composition affects classifier performance characteristics, Related to Figure 3

(A) Schematic illustrating how an AF-M prediction is converted into a numeric feature representation that can be fed into a random forest machine learning classifier. A random forest is trained by giving it example data with features along with the desired label/class. During training, the forest builds many independent decision trees out of random feature subsets, with each tree attempting to assign the correct label (0 or 1) to the data. Once trained, new instances to be predicted are passed through all the trees, with each tree routing the pair through all its decision layers until it hits a terminal node, where it is assigned a 0 or 1. This final binary vote is tallied across all trees and averaged to produce a final ensemble score. When working effectively, the classifier produces low scores for false pairs and higher scores for true pairs, but for complex data, this separation is generally not perfect. **(B)** A structural classifier was trained only data from ($RefSet^{XLMS}$) as positive examples and ($RefSet^{XLMSR}$) as negative examples. The resulting classifier was then applied on pairs excluded from training from three datasets and the resulting classifier scores were plotted as histograms. **(C)** Same as (A) but the negative examples were expanded to include the ($RefSet^{Random}$) **(D)** Same as (B), but the negative examples were expanded to include the ($RefSet^{Decoy}$). **(E)** Schematic illustrating how we converted the N:P ratio in the human binary interactome into the N:P ratio that would be observed in an unbiased AF-M screen. We assume 1.5 million TPs and 198.5 million TNs in the human proteome. Based on an AF-M rate of ~40% in identifying true positives (Figure 2E and accompanying results section), the 1.5 million proteomic TPs would produce 600,000 "hits." Analogously, because only 25% of random negative pairs satisfy the contact (C+) criterion (Figure 2A), 198.5 million negatives would yield ~50 million contact positive (C+) TN pairs. Dividing 50 million by 600,000 yields an apparent N:P ratio of ~80:1.

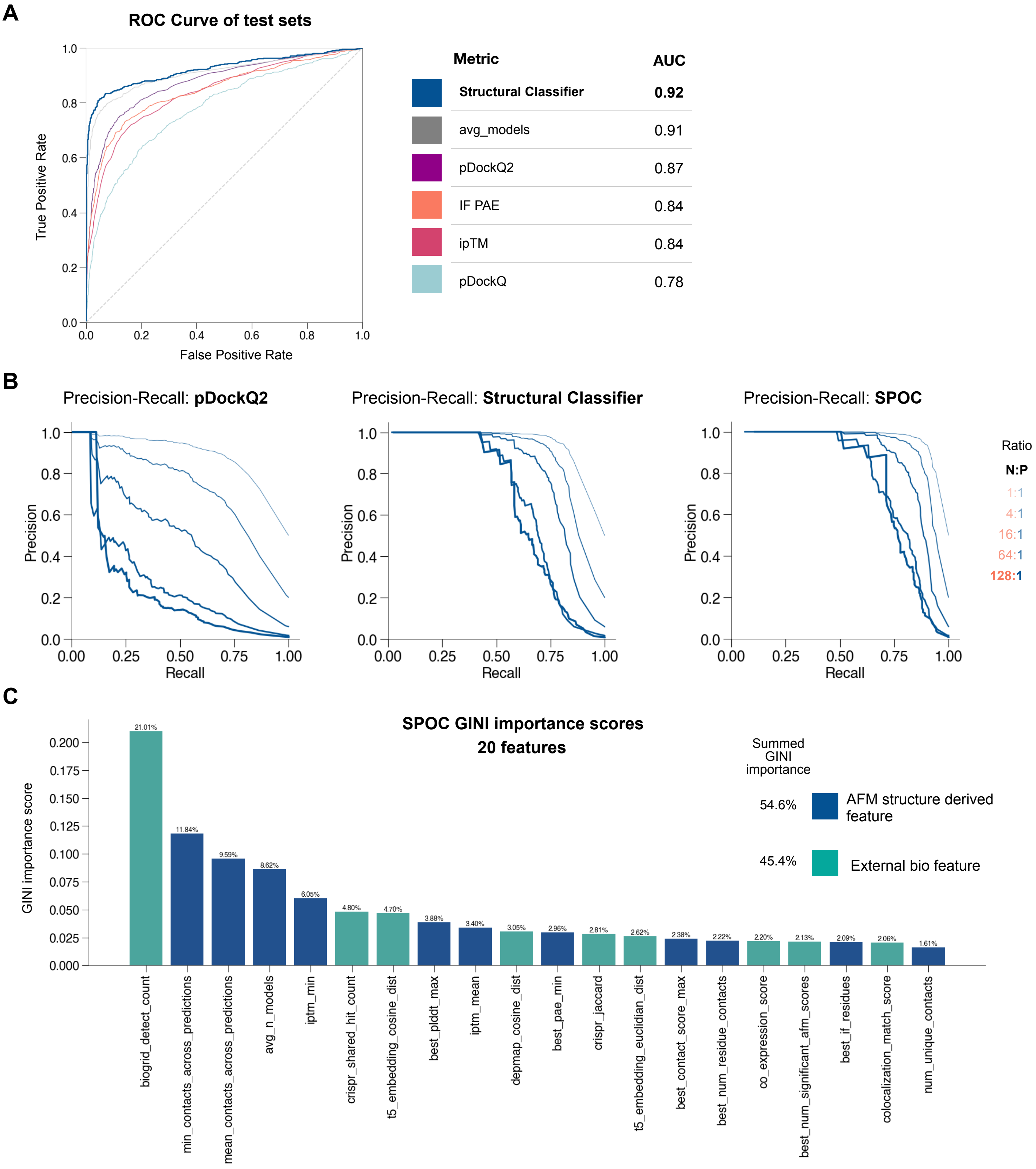

Figure S4. The SPOC classifier for assessing binary predictions, Related to Figure 4

Figure S4. The SPOC classifier for assessing binary predictions, Related to Figure 4

(A) AUC (Area Under the Curve) values for ROC (Receiver Operating Characteristic) curves for the structural classifier and other metrics. TN is ($RefSet^{Random}$ + $RefSet^{XLMSR}$ + $RefSet^{Decoy}$); TP set is ($RefSet^{XLMS}$+$RefSet^{PDB}$). The ROC curve shows the True Positive Rate (y) and False positive rate (x) as a function of selected threshold (which is not explicitly shown on the graph). The stringency increases (higher threshold) moving from the top right to the bottom left of the graph. The better a discriminator can separate false examples from true examples, the more its ROC curve shifts to the upper left, resulting in higher AUC values. **(B)** Precision recall (PR) plots show how recall TP/(TP+FN) and precision TP/(TP+FP) changes as a function of threshold. The best discriminators produce PR curves that tend toward the upper right area of the plot as they recover a high percentage of true examples without including too many false examples. In the plots shown here, SPOC exhibits the best performance, as measured by its curves staying closest to the upper right even as the N:P ratio in the test data increases. N set is ($RefSet^{Random}$ +$RefSet^{XLMSR}$ + $RefSet^{Decoy}$); P set is ($RefSet^{XLMS}$+$RefSet^{PDB}$). **(C)** Histogram of GINI importances. Training a random forest involves constructing decision trees to achieve optimal data splits. All trees record the features they used and how useful they were for sorting data into the correct categories. Splitting usefulness is quantified for every feature via a metric called GINI importance. A higher score indicates that a feature is more helpful in guiding predictions. Plotting GINI importances for SPOC reveals that many features drove its performance.

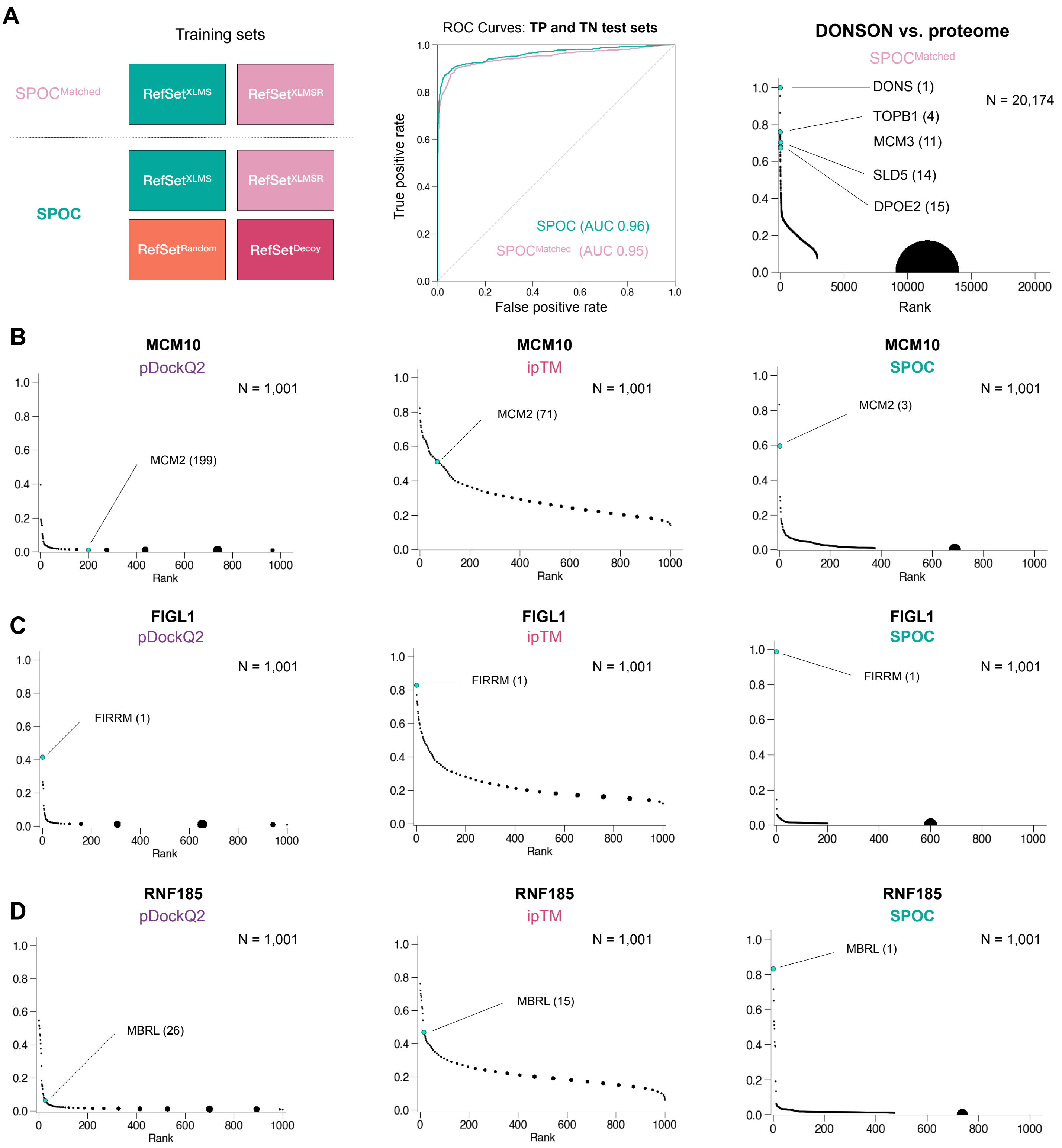

Figure S5. Comparing ranking performance of different metrics, Related to Figure 5

(A) Comparing the performance of SPOC vs SPOC^{Matched} using ROC curves and an example of proteome wide ranking experiment with the DONSON dataset. **(B)** The MCM10/MCM2 (Nucleus) true pair was embedded in 1000 random pairs involving MCM10, and the AF-M predictions for each pair were ranked using three different metrics. **(C)** The FIGL1-FIRRM (Nucleus) true pair was embedded in 1000 random pairs involving FIGL1, and the AF-M predictions for each pair were ranked using three different metrics. **(D)** The RNF185-MBRL (ER membrane) true pair was embedded in 1000 random pairs involving RNF185, and the AF-M predictions for each pair were ranked using three different metrics.

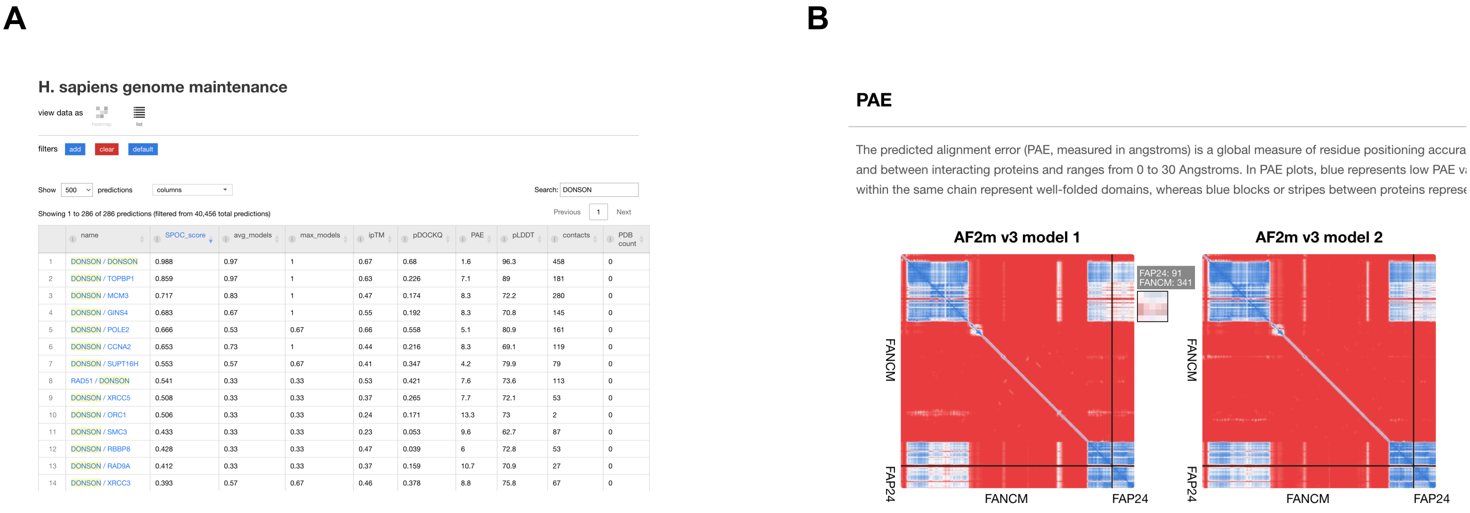

	name	SPOC_score	avg_models	max_models	ipTM	pDOCKQ	PAE	pLDDT	contacts	PDB count
1	DONSON / DONSON	0.988	0.97	1	0.67	0.68	1.6	96.3	458	0
2	DONSON / TOPBP1	0.859	0.97	1	0.63	0.226	7.1	89	181	0
3	DONSON / MCM3	0.717	0.83	1	0.47	0.174	8.3	72.2	280	0
4	DONSON / GINS4	0.683	0.67	1	0.55	0.192	8.3	70.8	145	0
5	DONSON / POLE2	0.666	0.53	0.67	0.66	0.558	5.1	80.9	161	0
6	DONSON / CCNA2	0.653	0.73	1	0.44	0.216	8.3	69.1	119	0
7	DONSON / SUPT16H	0.553	0.57	0.67	0.41	0.347	4.2	79.9	79	0
8	RAD51 / DONSON	0.541	0.33	0.33	0.53	0.421	7.6	73.6	113	0
9	DONSON / XRCC5	0.508	0.33	0.33	0.37	0.265	7.7	72.1	53	0
10	DONSON / ORC1	0.506	0.33	0.33	0.24	0.171	13.3	73	2	0
11	DONSON / SMC3	0.433	0.33	0.33	0.23	0.053	9.6	62.7	87	0
12	DONSON / RBBP8	0.428	0.33	0.33	0.47	0.039	6	72.8	53	0
13	DONSON / RAD9A	0.412	0.33	0.33	0.37	0.159	10.7	70.9	27	0
14	DONSON / XRCC3	0.393	0.57	0.67	0.46	0.378	8.8	75.8	67	0

Figure S6. A web portal for viewing and analyzing AF-M predictions, Related to Figure 6

(A) Typing a protein name into the search field on the matrix page of predictomes.org retrieves an interactive list of a protein's hits prioritized by SPOC score. Users can highlight specific rows for visual reference and retrieval. Clicking a row routes the user to the protein's information page. **(B)** Interactive PAE plots that describe the relative distance error (Å) between any two residues in the predicted complex. Hovering on the plots brings up a smaller view that displays the residue coordinates as well as a zoomed in view of the PAEs near the area of interest.

A

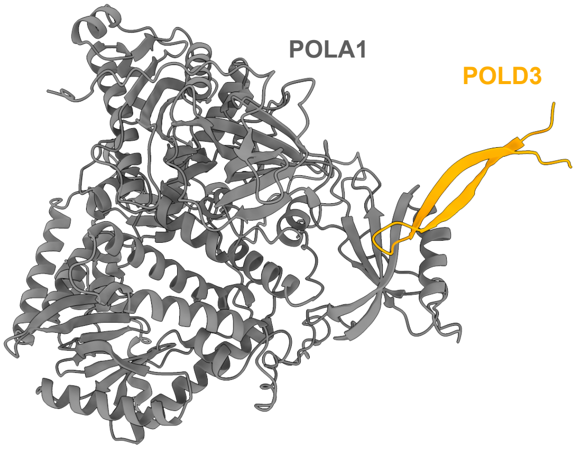

Binary AF-M prediction

B

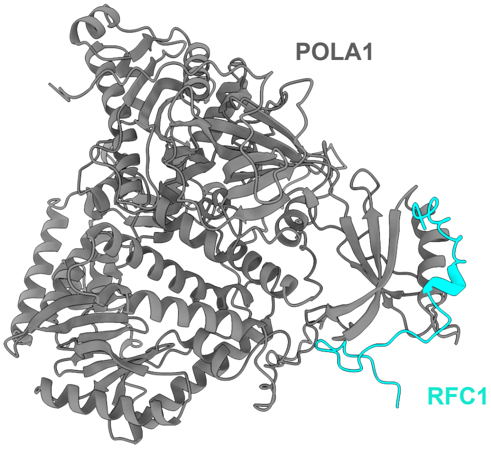

Binary AF-M prediction

Figure S7. Binary AF-M predictions, Related to Figure 7

(A) AF-M prediction of the human POLA1-POLD3 complex. The orientation of POLA1 is exactly as in Figure 7B. See Table S5 for confidence metrics. **(B)** AF-M prediction of the human POLA1-RFC1 complex. The orientation of POLA1 is exactly as in Figure 7B. The interaction between RFC1 and POLA1 is less extensive than in the presence of POLD3, as seen in Figure 7B. See Table S5 for confidence metrics.

Supplementary References:

1. Chavez, J.D., Schweppe, D.K., Eng, J.K., Zheng, C., Taipale, A., Zhang, Y., Takara, K., and Bruce, J.E. (2015). Quantitative interactome analysis reveals a chemoresistant edgotype. Nature Communications 2015 6:1 *6*, 1–12. https://doi.org/10.1038/ncomms8928.
2. Liu, F., Lössl, P., Scheltema, R., Viner, R., and Heck, A.J.R. (2017). Optimized fragmentation schemes and data analysis strategies for proteome-wide cross-link identification. Nature Communications 2017 8:1 *8*, 1–8. https://doi.org/10.1038/ncomms15473.
3. Fasci, D., Ingen, H. Van, Scheltema, R.A., and Heck, A.J.R. (2018). Histone Interaction Landscapes Visualized by Crosslinking Mass Spectrometry in Intact Cell Nuclei. Molecular & Cellular Proteomics *17*, 2018–2033. https://doi.org/10.1074/MCP.RA118.000924.
4. Liu, F., Lössl, P., Rabbitts, B.M., Balaban, R.S., and Heck, A.J.R. (2018). The interactome of intact mitochondria by cross-linking mass spectrometry provides evidence for coexisting respiratory supercomplexes. Molecular & Cellular Proteomics *17*, 216–232. https://doi.org/10.1074/MCP.RA117.000470.
5. Ryl, P.S.J., Bohlke-Schneider, M., Lenz, S., Fischer, L., Budzinski, L., Stuiver, M., Mendes, M.M.L., Sinn, L., O'Reilly, F.J., and Rappsilber, J. (2020). In Situ Structural Restraints from Cross-Linking Mass Spectrometry in Human Mitochondria. J Proteome Res *19*, 327–336. https://doi.org/10.1021/ACS.JPROTEOME.9B00541/ASSET/IMAGES/LARGE/PR9B00541_0003.JPEG.
6. Mendes, M.L., Fischer, L., Chen, Z.A., Barbon, M., O'Reilly, F.J., Giese, S.H., Bohlke-Schneider, M., Belsom, A., Dau, T., Combe, C.W., et al. (2019). An integrated workflow for crosslinking mass spectrometry. Mol Syst Biol *15*. https://doi.org/10.15252/MSB.20198994/SUPPL_FILE/MSB198994-SUP-0006-DATASETEV4.XLSX.
7. Ser, Z., Cifani, P., and Kentsis, A. (2019). Optimized Cross-Linking Mass Spectrometry for in Situ Interaction Proteomics. J Proteome Res *18*, 2545–2558. https://doi.org/10.1021/ACS.JPROTEOME.9B00085/SUPPL_FILE/PR9B00085_SI_002.XLSX.
8. Tayri-Wilk, T., Slavin, M., Zamel, J., Blass, A., Cohen, S., Motzik, A., Sun, X., Shalev, D.E., Ram, O., and Kalisman, N. (2020). Mass spectrometry reveals the chemistry of formaldehyde cross-linking in structured proteins. Nature Communications 2020 11:1 *11*, 1–9. https://doi.org/10.1038/s41467-020-16935-w.
9. Gonzalez-Lozano, M.A., Koopmans, F., Sullivan, P.F., Protze, J., Krause, G., Verhage, M., Li, K.W., Liu, F., and Smit, A.B. (2020). Stitching the synapse: Cross-linking mass spectrometry into resolving synaptic protein interactions. Sci Adv *6*. https://doi.org/10.1126/SCIADV.AAX5783/SUPPL_FILE/AAX5783_TABLE_S4.XLSX.
10. Wheat, A., Yu, C., Wang, X., Burke, A.M., Chemmama, I.E., Kaake, R.M., Baker, P., Rychnovsky, S.D., Yang, J., and Huang, L. (2021). Protein interaction landscapes revealed by advanced in vivo cross-linking-mass spectrometry. Proc Natl Acad Sci U S A *118*, e2023360118. https://doi.org/10.1073/PNAS.2023360118/SUPPL_FILE/PNAS.2023360118.SD08.XLSX.
11. Ihling, C.H., Piersimoni, L., Kipping, M., and Sinz, A. (2021). Cross-Linking/Mass Spectrometry Combined with Ion Mobility on a timsTOF Pro Instrument for Structural Proteomics. Anal Chem *93*, 11442–11450. https://doi.org/10.1021/ACS.ANALCHEM.1C01317/ASSET/IMAGES/LARGE/AC1C01317_0007.JPEG.
12. Jiao, F., Yu, C., Wheat, A., Wang, X., Rychnovsky, S.D., and Huang, L. (2022). Two-Dimensional Fractionation Method for Proteome-Wide Cross-Linking Mass Spectrometry Analysis. Anal Chem *94*, 4236–4242. https://doi.org/10.1021/ACS.ANALCHEM.1C04485/SUPPL_FILE/AC1C04485_SI_005.XLSX.
13. An, Y., Zhao, Q., Gong, Z., Zhao, L., Li, Y., Liang, Z., Zou, P., Zhang, Y., and Zhang, L. (2022). Suborganelle-Specific Protein Complex Analysis Enabled by in Vivo Cross-Linking Coupled with Proximal Labeling. Anal Chem. https://doi.org/10.1021/ACS.ANALCHEM.2C01637/SUPPL_FILE/AC2C01637_SI_002.XLSX.
14. Gao, H., Zhao, L., Zhong, B., Zhang, B., Gong, Z., Zhao, B., Liu, Y., Zhao, Q., Zhang, L., and Zhang, Y. (2022). In-Depth in Vivo Crosslinking in Minutes by a Compact, Membrane-Permeable,

and Alkynyl-Enrichable Crosslinker. Anal Chem *94*, 7551–7558. https://doi.org/10.1021/ACS.ANALCHEM.2C00335/SUPPL_FILE/AC2C00335_SI_004.XLSX.
15. Singh, J., Elhabashy, H., Muthukottiappan, P., Stepath, M., Eisenacher, M., Kohlbacher, O., Gieselmann, V., and Winter, D. (2022). Cross-linking of the endolysosomal system reveals potential flotillin structures and cargo. Nature Communications 2022 13:1 *13*, 1–18. https://doi.org/10.1038/s41467-022-33951-0.
16. Bartolec, T.K., Vázquez-Campos, X., Norman, A., Luong, C., Johnson, M., Payne, R.J., Wilkins, M.R., Mackay, J.P., and Low, J.K.K. (2023). Cross-linking mass spectrometry discovers, evaluates, and corroborates structures and protein–protein interactions in the human cell. Proc Natl Acad Sci U S A *120*, e2219418120. https://doi.org/10.1073/PNAS.2219418120/SUPPL_FILE/PNAS.2219418120.SD07.XLSX.
17. Jiao, F., Salituro, L.J., Yu, C., Gutierrez, C.B., Rychnovsky, S.D., and Huang, L. (2023). Exploring an Alternative Cysteine-Reactive Chemistry to Enable Proteome-Wide PPI Analysis by Cross-Linking Mass Spectrometry. Anal Chem *95*, 2532–2539. https://doi.org/10.1021/ACS.ANALCHEM.2C04986/SUPPL_FILE/AC2C04986_SI_005.XLSX.
18. Chen, J., Zhao, Q., Gao, H., Zhao, L., Chu, H., Shan, Y., Liang, Z., Zhang, Y., and Zhang, L. (2023). A Glycosidic-Bond-Based Mass-Spectrometry-Cleavable Cross-linker Enables In Vivo Cross-linking for Protein Complex Analysis. Angewandte Chemie International Edition *62*, e202212860. https://doi.org/10.1002/ANIE.202212860.
19. Zhang, B., Gao, H., Gong, Z., Zhao, L., Zhong, B., Sui, Z., Liang, Z., Zhang, Y., Zhao, Q., and Zhang, L. (2023). Improved Cross-Linking Coverage for Protein Complexes Containing Low Levels of Lysine by Using an Enrichable Photo-Cross-Linker. Anal Chem *95*, 9445–9452. https://doi.org/10.1021/ACS.ANALCHEM.2C05020/SUPPL_FILE/AC2C05020_SI_002.XLSX.
20. Chen, Y., Zhou, W., Xia, Y., Zhang, W., Zhao, Q., Li, X., Gao, H., Liang, Z., Ma, G., Yang, K., et al. (2023). Targeted cross-linker delivery for the in situ mapping of protein conformations and interactions in mitochondria. Nature Communications 2023 14:1 *14*, 1–16. https://doi.org/10.1038/s41467-023-39485-3.
21. Bryant, P., Pozzati, G., and Elofsson, A. (2022). Improved prediction of protein-protein interactions using AlphaFold2. Nature Communications 2022 13:1 *13*, 1–11. https://doi.org/10.1038/s41467-022-28865-w.
22. Zhu, W., Shenoy, A., Kundrotas, P., and Elofsson, A. (2023). Evaluation of AlphaFold-Multimer prediction on multi-chain protein complexes. Bioinformatics *39*. https://doi.org/10.1093/BIOINFORMATICS/BTAD424.
